# Impact of inflammation on brain subcellular energetics in anesthetized rats

**DOI:** 10.1186/s12868-019-0514-8

**Published:** 2019-07-15

**Authors:** Robert H. Thiele, Hari P. Osuru, Umadevi Paila, Keita Ikeda, Zhiyi Zuo

**Affiliations:** 1 Department of Anesthesiology, University of Virginia School of Medicine, P.O. Box 800710, Charlottesville, VA 22908-0710 USA; 2 Center for Public Health Genomics, University of Virginia School of Medicine, Charlottesville, USA

**Keywords:** Cecal ligation and puncture, Anaerobic metabolism, Subcellular energetics, Cytochrome aa_3_, Hypoxia-inducible factor 1

## Abstract

**Background:**

Emerging data suggests that volatile anesthetic agents may have organ protection properties in the setting of critical illness. The purpose of this study was to better understand the effect of inflammation on cerebral subcellular energetics in animals exposed to two different anesthetic agents—a GABA agonist (propofol) and a volatile agent (isoflurane).

**Results:**

Forty-eight Sprague–Dawley rats were anesthetized with isoflurane or propofol. In each group, rats were randomized to celiotomy and closure (sham) or cecal ligation and puncture (inflammation [sepsis model]) for 8 h. Brain tissue oxygen saturation and the oxidation state of cytochrome aa_3_ were measured. Brain tissue was extracted using the freeze-blow technique. All rats experienced progressive increases in tissue oxygenation and cytochrome aa_3_ reduction over time. Inflammation had no impact on cytochrome aa_3_, but isoflurane caused significant cytochrome aa_3_ reduction. During isoflurane (not propofol) anesthesia, inflammation led to an increase in lactate (+ 0.64 vs. − 0.80 mEq/L, *p* = 0.0061). There were no differences in ADP:ATP ratios between groups. In the isoflurane (not propofol) group, inflammation increased the expression of hypoxia-inducible factor-1α (62%, *p* = 0.0012), heme oxygenase-1 (67%, *p* = 0.0011), and inducible nitric oxide synthase (31%, *p* = 0.023) in the brain. Animals exposed to inflammation and isoflurane (but not propofol) exhibited increased expression of protein carbonyls (9.2 vs. 7.0 nM/mg protein, *p* = 0.0050) and *S*-nitrosylation (49%, *p* = 0.045) in the brain. RNA sequencing identified an increase in heat shock protein 90 and NF-κβ inhibitor mRNA in the inflammation/isoflurane group.

**Conclusions:**

In the setting of inflammation, rats exposed to isoflurane show increased hypoxia-inducible factor-1α expression despite a lack of hypoxia, increased oxidative stress in the brain, and increased serum lactate, all of which suggest a relative increase in anaerobic metabolism compared to propofol. Differences in oxidative stress as well as heat shock protein 90 and NF-κβ inhibitor may account for the differential expression of cerebral hypoxia-inducible factor-1α during inflammation.

**Electronic supplementary material:**

The online version of this article (10.1186/s12868-019-0514-8) contains supplementary material, which is available to authorized users.

## Background

Commonly used anesthetic agents have varying organ-protective properties that have the potential to attenuate the impact of stressors such as ischemia, reperfusion, and inflammation. Pre-exposure with volatile anesthetic agents (VAAs) has produced organ-protective effects in several settings, including myocardial infarction, cerebral ischemia, and ischemia–reperfusion [1]. This effect is referred to as anesthetic preconditioning (APC) and is mechanistically related to ischemic preconditioning (IPC) [2]. While primarily studied in stroke and myocardial infarction models, isoflurane pre-treatment has been shown to preserve hemodynamics in experimental sepsis [3, 4], and recent comparisons between VAAs and GABA agonists in animals suggest a mortality benefit in animals exposed to VAAs [5, 6]. A more recent pilot study comparing VAAs to GABA agonists in humans with ARDS showed improved oxygenation and reduced endothelial injury and inflammation in the VAA group [7].

While VAAs are not typically used in the ICU setting, if the organ-protective effects observed in the operating room can be extended to pathologies commonly seen in the ICU (e.g. sepsis), VAAs may offer a new treatment modality for some critical illnesses. The recent development of a portable vaporizer that can be used with any ICU ventilator [7] may facilitate the introduction of VAAs into the ICU setting.

Mitochondria play a central role in the metabolic activity of both healthy and diseased eukaryotic organisms, including during sepsis, primarily through their central role in oxidative phosphorylation. This efficient process produces ATP as electrons pass down the electron transport chain (ETC), generating a proton gradient which drives ATP synthase [8]. Oxidative phosphorylation can be altered by changes in mitochondrial oxygen supply, the availability of electron donors from the tricyclic acid (TCA) cycle, or direct inhibition of the cytochromes [8]. The impact of sepsis on subcellular energetics and metabolism is still not clear [8].

Both VAAs and GABA agonists are known to affect subcellular metabolism—VAAs inhibit Complex I [9] and lead to anaerobic metabolism and lactate production [10]. Propofol does not appear to inhibit Complex I [11] but may inhibit Complex II [12]. Differences in metabolism between sedating agents may explain their differential effects on both animal models and humans exposed to sepsis. The primary purpose of this study was to compare the impact of inflammation on brain metabolism (oxidation state of cytochrome aa_3_ [Cyt_OX_] derived from broadband near infrared spectroscopy [NIRS]) in an established animal model of sepsis (cecal ligation and puncture [CLP]) using two different anesthetic regimens—VAAs (isoflurane) and GABA agonists (propofol). We used the CLP model because it is thought to be a more realistic model of human sepsis than, for instance, lipopolysaccharide infusion [13]. Secondary outcomes included changes in ADP:ATP ratio, oxidative stress, and expression of proteins involved in subcellular energetics.

## Results

### Markers of illness severity

**Fig. 1:**
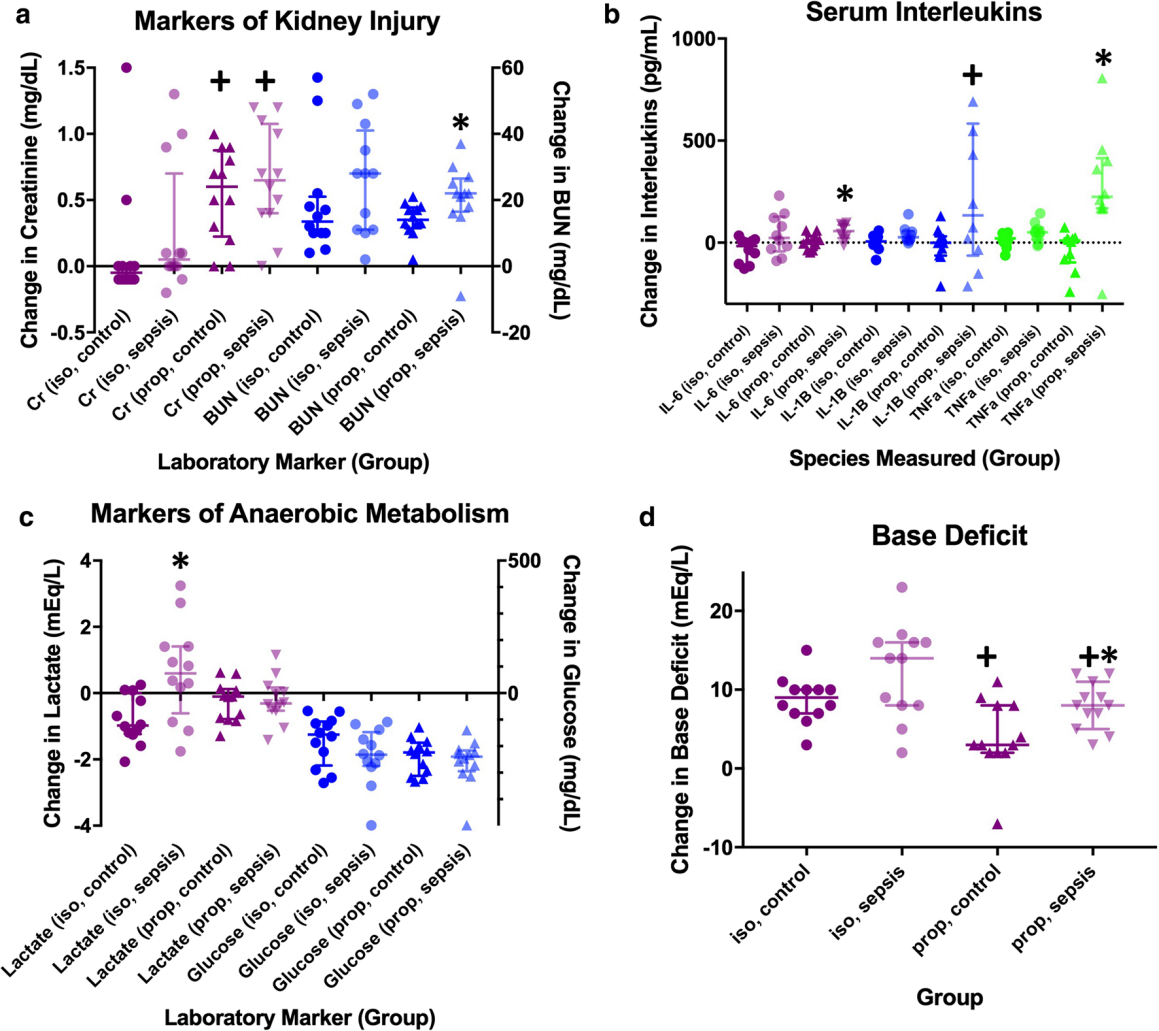
Changes in laboratory values taken from serum both before and after the surgical procedure in controls as well as animals subjected to sepsis. **a** Markers of kidney injury (bars represent median + interquartile range [IQR]); **b** inflammatory markers (median + IQR); **c** markers of anaerobic metabolism (median + IQR); **d** base deficit (mean + 95% confidence interval). *Denotes a statistically significant difference between animals exposed to sepsis and controls. ^+^denotes a statistically significant difference between animals anesthetized with isoflurane and propofol

Blood pressure, intravenous fluid, lactic acid, base deficit, glucose, blood urea nitrogen (BUN), creatinine, and serum interleukin data are presented in Table 1 and Fig. 1. All 48 animals completed the study and were analyzed, and there were no adverse events. In both the isoflurane and propofol groups, animals exposed to CLP required more fluid, had lower mean arterial pressure after 8 h, higher base deficits, and increase IL-6, IL-1β, and TNF-α at 8 h as compared to sham controls.

**Table 1:** Use of intravenous fluids, mean arterial pressure, and changes in laboratory values

Variable	Isoflurane groups					Propofol groups				
	Sham (isoflurane)		CLP (isoflurane)		Sham vs. CLP	Sham (propofol)		CLP (propofol)		Sham vs. CLP
	Mean	SD	Mean	SD	*p* value	Mean	SD	Mean	SD	*p* value
Fluids given (mL/kg)	6.91	2.11	13.21	3.32	*0.004*	11.89	6.21	20.85	5.71	*0.001*
MAP (start)	95.86	8.69	95.92	13.17	0.507	90.71	19.37	75.44	16.34	0.049
MAP (end)	102.38	8.71	77.06	23.08	*0.001*	82.35	12.78	67.23	16.61	*0.020*
MAP change	6.53	5.51	− 18.85	25.51	*0.002*	− 8.36	21.98	− 8.22	18.56	0.987
Lactate (start)	2.68	0.82	1.85	0.64	*0.007*	1.11	0.49	1.06	0.55	0.789
Lactate (end)	1.88	0.56	2.48	1.19	0.125	0.85	0.30	0.85	0.38	0.972
Lactate (change)	− 0.80	0.72	0.64	1.48	*0.006*	− 0.26	0.60	− 0.21	0.69	0.843
Lactate (clearance)	27.58	22.71	− 50.88	84.40	*0.005*	− 1.20	77.69	− 3.72	86.06	0.931
BE (start)	4.92	4.14	4.75	4.14	0.922	7.42	2.81	6.50	2.11	0.197
BE (end)	− 3.83	4.11	− 7.58	4.01	*0.034*	3.42	3.23	− 1.42	3.90	*0.003*
BE change	− 8.75	2.99	− 12.33	5.96	0.076	− 4.00	4.69	− 7.92	2.94	*0.023*
Glucose (start)	379.33	85.57	396.75	86.98	0.626	375.75	50.83	373.17	92.47	0.665
Glucose (end)	200.33	43.81	159.42	85.93	0.156	136.17	25.21	113.42	22.50	*0.019*
Glucose change	− 179.00	94.24	− 237.33	108.88	0.174	− 239.58	66.84	− 259.75	88.57	0.686
Anesth. dose	1.50	0.00	1.40	0.13	*0.016*	406.67	167.46	327.08	126.39	0.202
Cr (start)	0.28	0.05	0.30	0.10	0.421	0.25	0.10	0.25	0.08	0.801
Cr (end)	0.39	0.48	0.57	0.52	0.088	0.79	0.34	0.91	0.40	0.450
Cr (change)	0.12	0.47	0.27	0.50	0.128	0.54	0.35	0.66	0.41	0.459
Cr (clearance)	− 38.89	155.59	− 94.86	183.41	0.121	− 242.36	160.08	− 290.28	200.56	0.524
BUN (start)	20.58	2.64	22.17	2.55	0.336	20.00	3.81	19.83	3.33	0.861
BUN (end)	39.75	17.49	48.25	17.28	0.244	34.08	6.26	40.33	10.71	*0.026*
BUN (change)	19.17	16.92	26.08	16.43	0.082	14.08	5.05	20.50	11.12	*0.008*
IL-6 (base)	145.00	50.79	146.05	33.92	0.957	67.12	22.87	51.04	24.82	0.104
IL-6 (8 h)	108.85	36.72	186.77	94.75	*0.026*	71.18	24.61	103.49	34.38	*0.027*
IL-6 difference	− 36.14	60.25	40.72	102.63	0.056	4.06	37.77	52.44	38.03	*0.011*
TNF-a (base)	11.32	22.62	15.90	19.74	0.635	18.27	85.50	32.29	200.92	0.733
TNF-a (8 h)	25.26	21.85	66.09	45.97	*0.021*	− 17.79	25.99	305.22	205.95	*0.000*
TNF-a difference	13.94	34.75	50.19	47.14	0.066	− 36.06	95.83	272.93	269.44	*0.003*
IL-1B (base)	98.90	74.58	114.38	38.48	0.567	323.29	106.47	354.79	75.78	0.456
IL-1B (8 h)	101.73	58.06	153.34	41.77	*0.035*	309.60	95.37	625.25	398.89	*0.026*
IL-1B difference	2.84	40.46	38.96	41.35	0.088	− 13.69	90.66	270.46	428.46	0.055

italic denotes a statistically significant difference between animals exposed to sepsis and controls

In rats anesthetized with isoflurane, inflammation led to an increase in lactate (+ 0.64 mEq/L vs. − 0.80 mEq/L, *p* = 0.0061), whereas in rats anesthetized with propofol, inflammation had no impact on the change in lactate (− 0.26 mEq/L vs. − 0.21 mEq/L, *p* = 0.84). After induction and establishment of arterial access (prior to sham surgery or CLP), rats anesthetized with isoflurane had significantly higher lactate values than rats anesthetized with propofol (2.13 vs. 1.0, *p* < 0.0001).

### Tissue oxygenation and cytochrome aa_3_ oxidation state (Cyt_OX_)

**Fig. 2:**
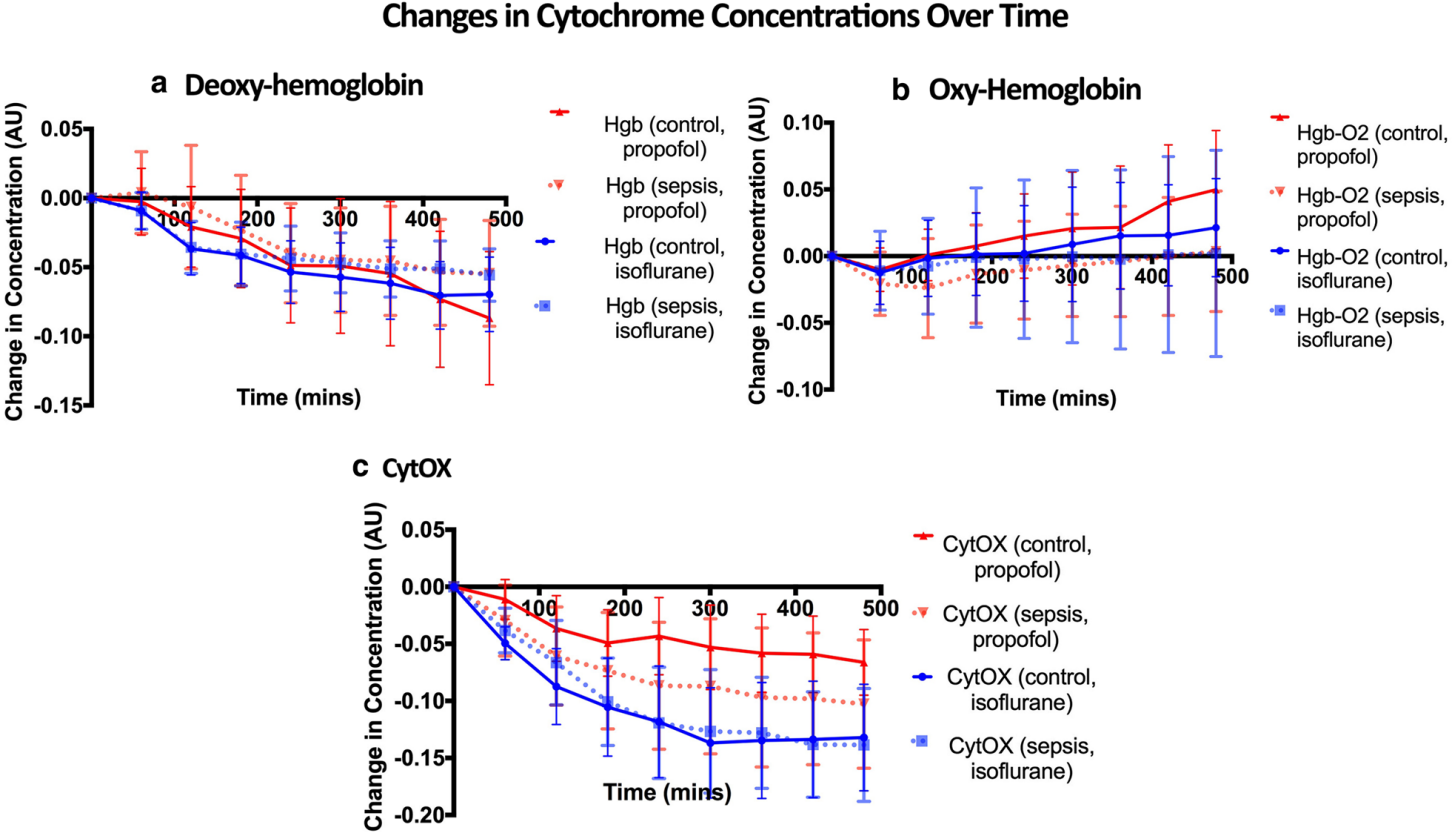
Relative concentration of deoxy-hemoglobin (**a**), oxy-hemoglobin (**b**), and Cyt_OX_ (c) for controls (solid line) and animals exposed to sepsis (dotted line), bounded by 95% confidence intervals. Based on a permutation F-test, neither deoxy-hemoglobin or oxyhemoglobin were affected by the anesthetic agent or sepsis, however the anesthetic agent (but not sepsis) did have a significant effect on Cyt_OX_

Rats anesthetized with both isoflurane and propofol experienced progressive increases in tissue oxygenation (decrease in deoxyhemoglobin, increases in oxyhemoglobin) and decreases in Cyt_OX_ over time (Fig. 2). Based on time series analysis (permutation F-test, see Additional file 1: Statistical Analysis of NIRS Data), neither hemoglobin or oxy-hemoglobin were affected by inflammation or the anesthetic modality. In contrast, anesthetic modality (*p* = 0.006) but not inflammation (*p* = 0.71) had a significant effect on Cyt_OX_.

### ADP:ATP ratio

**Fig. 3:**
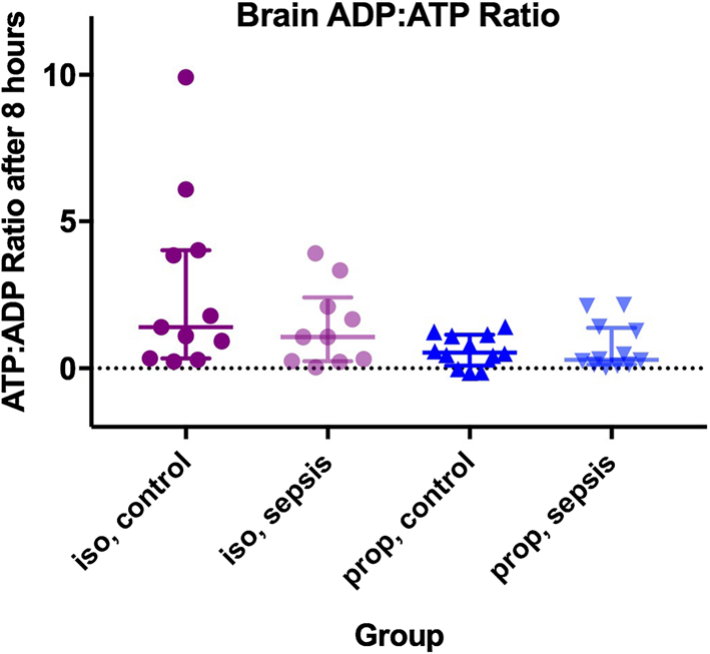
Brain ADP:ATP ratio (bars represent median + IQR) in controls and animals exposed to sepsis and anesthetized with both isoflurane and propofol. Supratentorial brain was instantaneously removed from live, anesthetized animals using the freeze-blow technique. There was no difference between groups

At the end of 8 h of anesthesia, ADP:ATP ratios in the brain were no different in septic rats than in controls, for animals exposed to both isoflurane (1.39 [0.33–4.0] vs. 1.06 [0.24–2.4], *p* = 0.282) and propofol (0.61 [0.27–0.95] vs. 0.715 [0.21–1.2], *p* = 0.714, Fig. 3).

### Protein expression

**Fig. 4:**
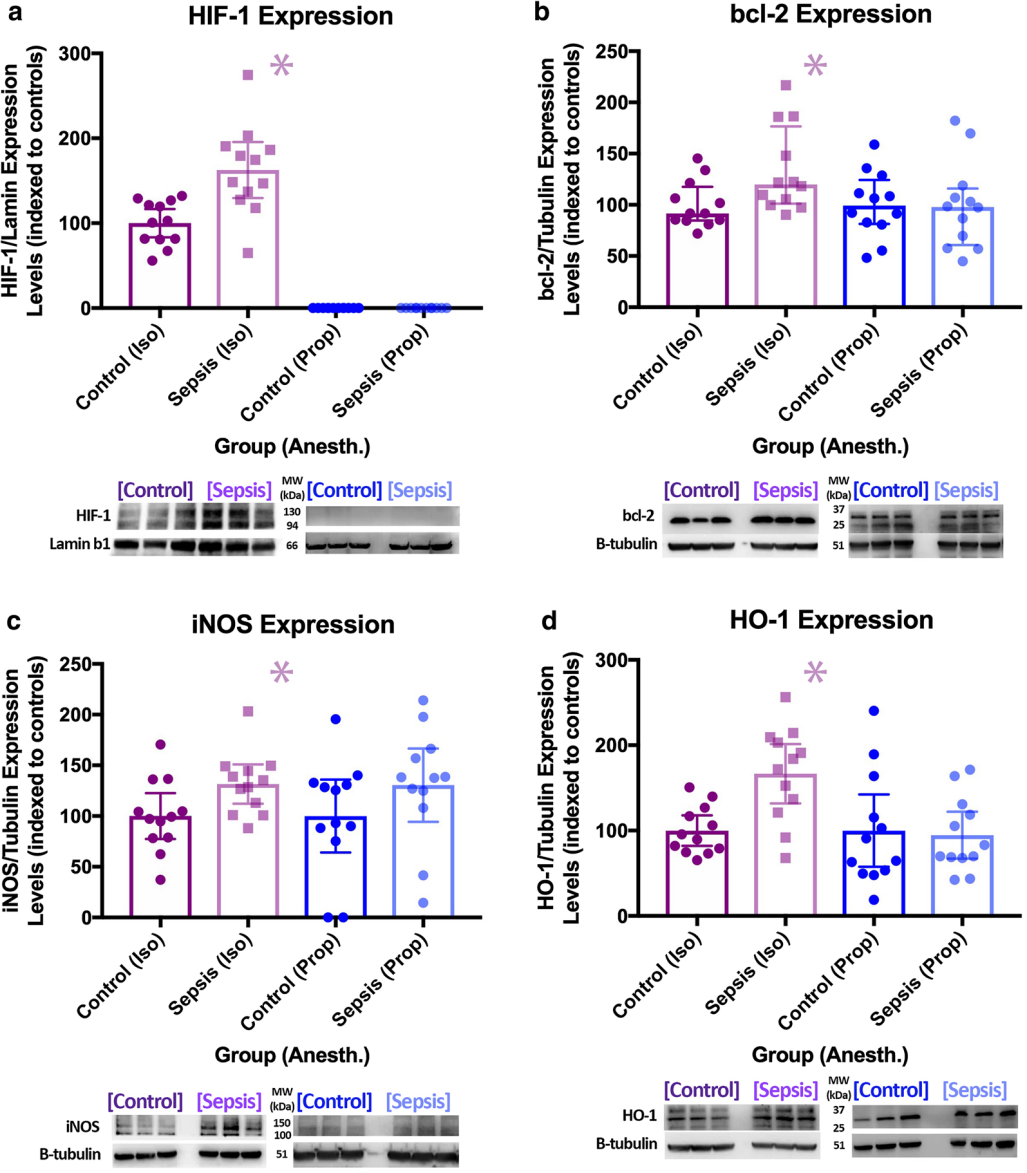
Protein expression data (upper) and representative raw data (lower) for animals anesthetized with isoflurane (bcl-2 [**a** median + IQR], HIF-1α [**b** mean + 95% confidence interval (CI)], HO-1 [**c** mean + 95% CI for isoflurane, median + IQR for propofol], iNOS [**d** mean + 95% CI for isoflurane, median + IQR for propofol) *denotes a statistically significant difference between animals exposed to sepsis and controls

In the isoflurane group, inflammation led to an increase in the expression of hypoxia-inducible factor-1 (HIF-1α, 62%, *p* = 0.0012), heme oxygenase-1 (HO-1, 67%, *p* = 0.0011), inducible nitric oxide synthase (iNOS, 31%, *p* = 0.023), and B cell lymphoma 2 (bcl-2, 34%, *p* = 0.022) in the brain. In animals anesthetized with propofol, there were no differences in protein expression between groups (Fig. 4).

### Oxidative stress

**Fig. 5:**
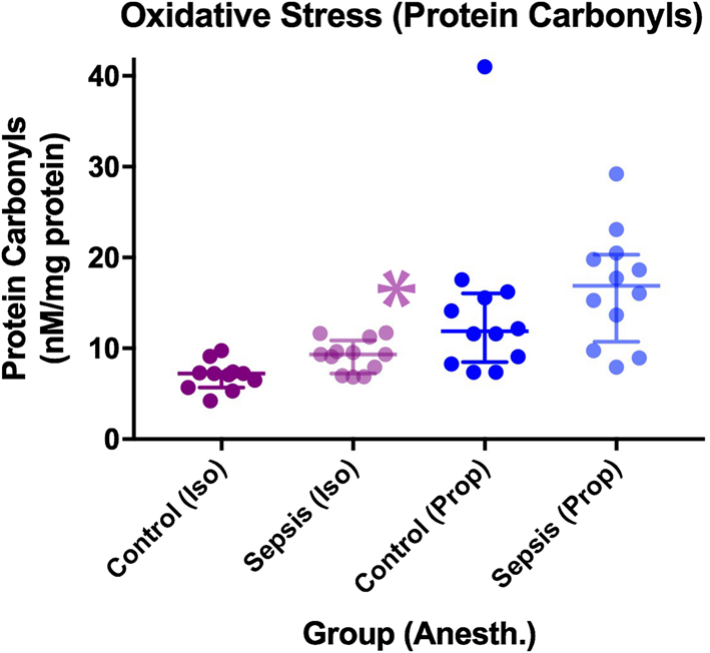
Differences in oxidative stress between groups as measured by protein carbonyls (median + IQR). *denotes a statistically significant difference between animals exposed to sepsis and controls

**Fig. 6:**
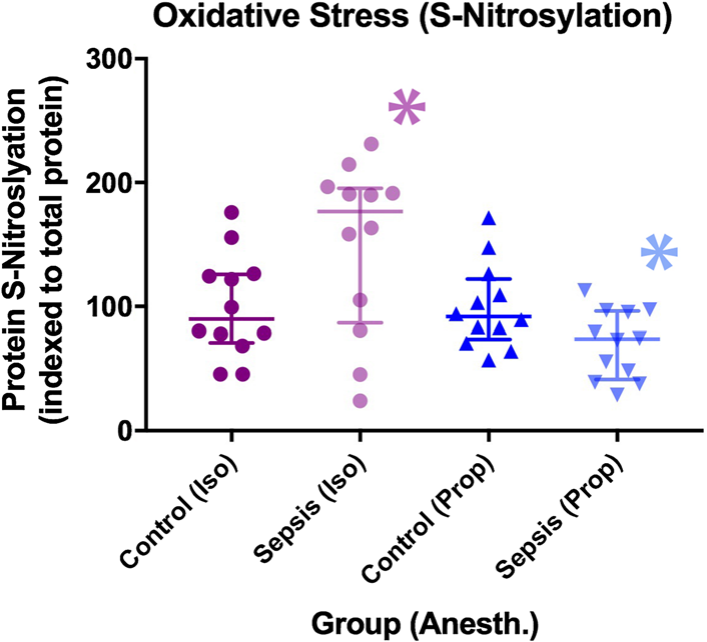
Differences in oxidative stress between groups as measured by *S*-nitrosylated protein (median + IQR). *denotes a statistically significant difference between animals exposed to sepsis and controls

Septic animals anesthetized with isoflurane exhibited increased expression of protein carbonyls (9.2 vs. 7.0 nM/mg protein, *p* = 0.0050) and *S*-nitrosylation (49%, *p* = 0.045) in the brain. In animals anesthetized with propofol, protein carbonyls were unchanged in the brain and *S*-nitrosylation was decreased (− 30%, *p* = 0.027, Figs. 5 and 6).

### Messenger RNA expression

Differentially expressed (on exposure to inflammation) genes related to metabolism, oxidative phosphorylation, oxidative stress, and cellular protection are presented in Table 2. For completeness, all statistically significant up and down-regulated genes identified by differential expression analysis of RNA sequencing are presented in Additional file 2: Table S1. In rats anesthetized with isoflurane, exposure to inflammation led to upregulation of 9 different heat shock protein (hsp) genes, upregulation of 7 ATPase-related genes, downregulation of 4 lipid-metabolism related genes, and down-regulation of 3 insulin-like growth factor (IGF)-1/IGF-1 binding protein-related genes. Adenosine A2a receptor and lactate dehydrogenase B were also upregulated in response to inflammation. In rats anesthetized with propofol, exposure to inflammation led to downregulation of 3 lipid-metabolism related genes, and down-regulation of 3 IGF-1/IBF binding protein-related genes. Additionally, NFKB inhibitor alpha, hypoxia inducible factor 3 subunit alpha, von Hippel-Lindau tumor suppressor, and lipopolysaccharide-induced TNF factor were also upregulated in response to inflammation.

**Table 2:**
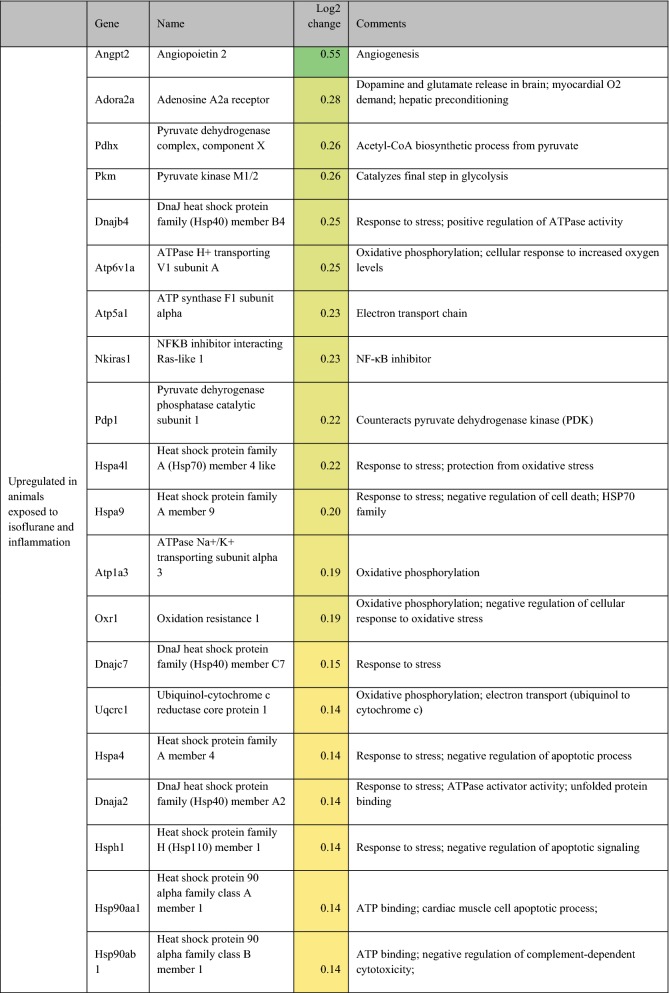

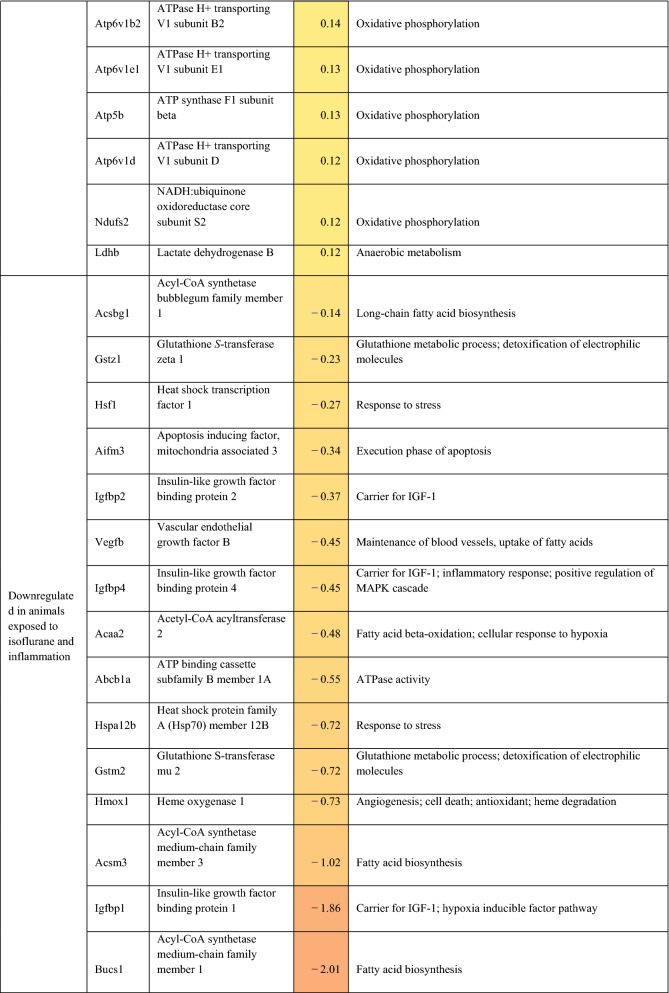

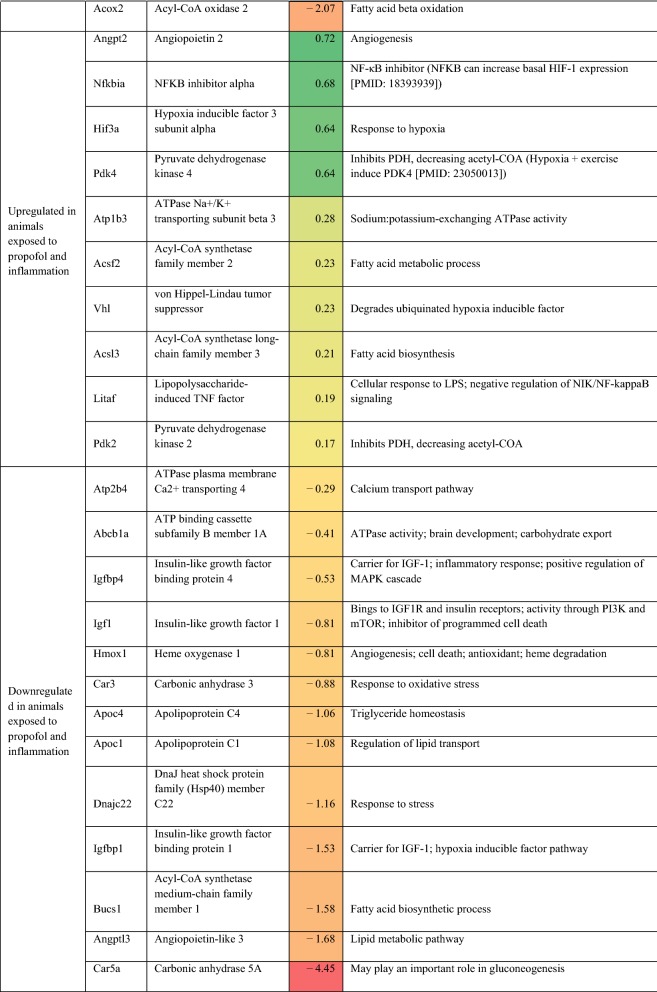
Potentially relevant genes either up or down-regulated in the setting of inflammation, as identified by in RNA sequencing

## Discussion

While not generally thought of as one of the primary organ systems affected by sepsis, the central nervous system is adversely, and in some cases permanently impacted by the dysregulated host response to infection [14]. Furthermore, sepsis is a known risk factor for long-term cognitive dysfunction in survivors of critical illness [15]. Thus, a better understanding of the impact of inflammation on the brain, and potential protective mechanisms, is needed.

**Fig. 7:**
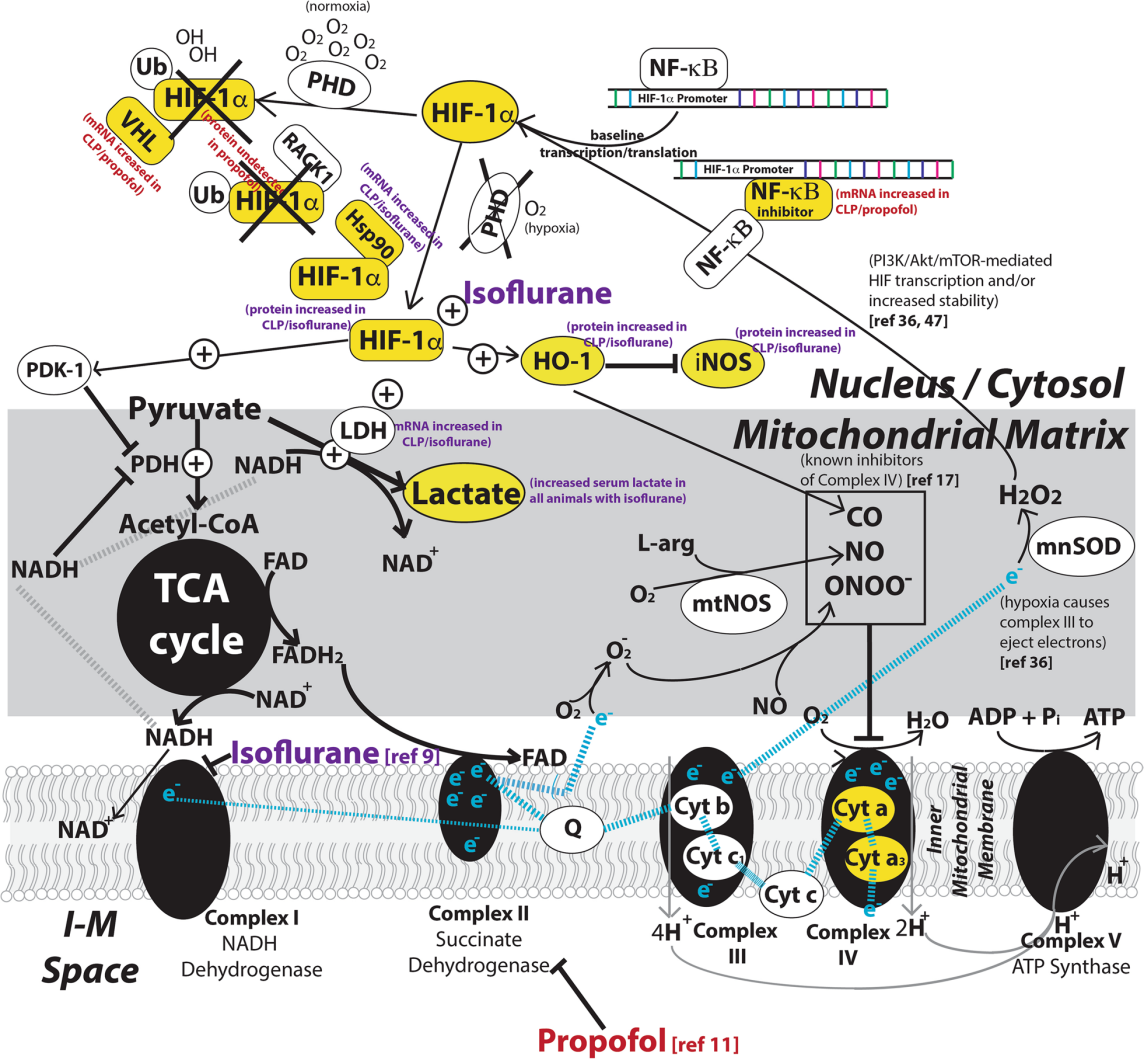
Potential mechanisms for our observations including references to other studies. Species measured in our study are marked in yellow. Propofol and isoflurane are known to inhibit Complex I and II of the ETC, respectively. Our data suggest that animals anesthetized with isoflurane, inflammation leads to increased expression of HIF-1α, HO-1, and iNOS, as well as an increase in Hsp90 mRNA and increased oxidative stress (the latter of which promotes through the PI3K/Akt/mTOR pathway). Isoflurane also causes an increase in lactate production as well as inhibition of Complex IV based on NIRS analysis. Our data also suggest that animals anesthetized with Propofol express no detectable HIF-1α, and in the setting of inflammation experience an increase in NF-κβ inhibitor, which prevents transcription of basal HIF-1α, as well as no detectable increase in oxidative stress

Emerging data suggest that VAAs favorably impact survival and organ dysfunction in both animals and humans exposed to inflammation and sepsis [5–7]. While the mechanisms of APC have been well-described, virtually all of the research on APC has focused on either myocardial or neurologic dysfunction following ischemia [16]. APC is complex and known to involve activation of the myocardial K_ATP_ channels, activation of protein kinase C, generation of oxidative species, as well as alterations in the mitochondrial permeability transition pore [16]. Metabolic differences in VAAs and GABA agonists are well-described [10, 17], but these studies do not include animals exposed to inflammation. Our study utilized an animal model of inflammation to better understand the role of different anesthetic agents on cellular metabolism and oxidative stress during critical illness. Our results, some of which are described schematically in Fig. 7, suggest the following.

### Isoflurane leads to anaerobic metabolism

The ability of VAAs to induce lactic acidosis has been described in multiple animals including humans [10, 18]. Prior to cecal ligation and puncture, rats in the isoflurane group exhibited higher lactate levels than rats in the propofol group (2.13 vs. 1.12 mEq/L, *p* < 0.0001). Interestingly, rats in the isoflurane group exhibited higher lactate levels 8 h after sham surgery (1.88 vs. 0.851 mEq/L, *p* < 0.0001) as well as after CLP (2.48 vs. 0.846 mEq/L, *p* = 0.0002) when compared to rats anesthetized with propofol.

Exposure of mitochondrial preparations to VAAs has revealed that halothane, isoflurane, and sevoflurane all inhibit Complex I of the ETC [9]. Inhibition of Complex I by isoflurane leads to an accumulation of NADH and pyruvate, while still allowing FADH to donate some electrons to enter the ETC via Complex II. NADH inhibits pyruvate dehydrogenase and is oxidized to NAD^+^ during the conversion of pyruvate to lactate via lactate dehydrogenase (LDH)—increased concentrations of pyruvate, coupled with pyruvate dehydrogenase inhibition would therefore be expected to increase the production of lactate. These effects are consistent with our observation of lactic acidosis in rats exposed to isoflurane.

While exposure of isolated mitochondria to isoflurane does not produce Complex IV (cytochrome aa_3_) inhibition, we observed a reduction in cytochrome aa_3_ measured in real time in our intact animal model (similar to what our laboratory has observed following injection of cyanide [19]). The source of this inhibition is not known, although candidate agents include nitric oxide, peroxynitrate, and carbon monoxide. Reactive species have been shown to directly inhibit Complex IV [20], as does carbon monoxide, a byproduct of the anti-oxidant protein HO-1. VAAs have been demonstrated to increase reactive oxidative species (ROS) production [16], inducing oxidative stress [21] as well as HO-1 expression [22].

### Isoflurane, not inflammation, inhibits the electron transport chain in the brain

We found no evidence that inflammation itself leads to ETC inhibition. Early studies suggesting that inflammation led to Cyt_OX_ reduction used discrete wavelength NIRS devices incapable of accurately measuring changes in Cyt_OX_ [23] and did not control for the anesthetic agent used. Early work using a 3 wavelength NIRS device suggested intravenous injection of *E. coli* leads to a rapid reduction in Cyt_OX_ [24]. Similar results were obtained by another group that injected *S. pneumonia* into rabbit cerebrospinal fluid (CSF) and measured Cyt_OX_. Both of these studies utilized volatile anesthetic agents. In contrast, Park et al. [25] injected *E. coli* into the CSF of newborn piglets anesthetized with thiopental, measured Cyt_OX_ and reported an *increase* in Cyt_OX_.

Our study differed from prior work in several respects. First, we used a live, polymicrobial infection model that is thought to be more reproducible and clinically relevant than direct injection of bacteria or endotoxin. Second, we used equipment that has been tested and proven to measure changes in Cyt_OX_ independent of changes in tissue oxygenation (StO_2_) [19]. Third, we measured Cyt_OX_ continuously for 8 h. Fourth, in dissecting the temporalis muscle, we were able to eliminate any potential interference from myoglobin in our measurements.

It may be that endotoxin does inhibit cytochrome aa_3_, but at higher concentrations than we were able to achieve with our 8-h CLP model. Measuring Cyt_OX_ accurately beyond 8 h is technically challenging as it requires the use of a stereotactic frame and general anesthesia for protracted periods of time. Although the time course of bacterial load after cecal ligation and puncture is not well described, evidence from both mouse and rat models suggests that colony forming units measured in the blood peak at approximately 6–12 h [26, 27].

### Early inflammation does not impact ADP:ATP ratio in the brain

Compared to controls, we observed no difference in the ADP:ATP ratio in the brain of rats exposed to inflammation for 8 h (for both the isoflurane and propofol groups). This is consistent with other published work. Synthesis of data from multiple animal models of varying severity and time points suggests that mitochondrial dysfunction causing energy failure requires approximately 24 h of inflammation and sepsis exposure [8] and similar results have been demonstrated in septic human surgical patients [28–31].

### Isoflurane induces and propofol attenuates cerebral oxidative stress in early inflammation

In animals anesthetized with isoflurane and exposed to 8 h of CLP, oxidative stress was detected in brain when measured with both protein carbonyls and *S*-nitrosylation. In contrast, animals anesthetized with propofol exhibited decreased oxidative stress in the brain as measured with *S*-nitrosylation (no difference in protein carbonyls). Propofol has previously been reported to offer some protection from oxidative stress in animal models of ischemia–reperfusion [12] and neuronal excitotoxicity [32].

### Inflammation leads to non-hypoxic HIF-1α upregulation in the brain

HIF-1α upregulation has been observed in the setting of inflammation [33–36] but these studies did not report tissue oxygenation. In the isoflurane group, we also observed HIF-1α upregulation in the brain, although we identified an *increase* in cerebral StO_2_. This suggests that HIF-1α upregulation in inflammation caused by a non-hypoxic stimulus.

ROS (which are produced in the setting of both hypoxia and hyperoxia) have been shown to be an essential component of HIF-1α stabilization and overexpression [37–39] and may explain other observations of non-hypoxic HIF-1α overexpression [40–43]. While we did not measure ROS explicitly, we also observed an inflammation-induced increase in iNOS expression, increased markers of oxidative stress, and an increase in the anti-oxidant protein HO-1 in our isoflurane group, all of which suggest that increased ROS production may be responsible for the observed difference in HIF-1α in animals exposed to isoflurane.

Interestingly, we could not detect any evidence of cerebral HIF-1α expression in our propofol groups (control and inflammation). HIF-1α is rapidly degraded by prolyl hydroxylases (PHD) and is not detectable in normal tissues unless PHDs are stabilized by lack of oxygen or the presence of oxidative stress. Our observations are consistent with previous literature that suggests propofol attenuates oxidative stress in the brain [12, 32] and inhibits HIF-1α expression in a dose-dependent fashion [44].

Additionally, we found evidence of increased Hsp90 mRNA expression in animals anesthetized with isoflurane and exposed to sepsis. Hsp90 competes with RACK1 to bind HIF-1α. RACK1 promotes ubiquitination (and subsequent degradation) of HIF-1α, and inhibition of Hsp90 is known to lead to HIF-1α degradation [45]. Increase Hsp90 expression may therefore represent a second non-hypoxic mechanism for increased HIF-1α expression in the setting of inflammation.

### Messenger RNA implications on organ protection

Analysis of mRNA expression suggests that isoflurane and propofol may produce differential metabolic responses as well as activate distinct organ protective pathways when animals are faced with an inflammatory stressor. In animals anesthetized with isoflurane, inflammation leads to upregulation of 9 distinct Hsp genes, which are known to be protective in a variety of stress states including sepsis [46]. Interestingly, this group also exhibited an increase in expression of lactate dehydrogenase (consistent with increased serum lactate) and adenosine A2a receptor mRNA, the latter of which is a known mediator of hepatic preconditioning [47].

In animals anesthetized with propofol, inflammation leads to upregulation of NF-κβ inhibitor α mRNA. NF-κβ increases basal HIF-1α expression [48], thus increased inhibitor expression might lead to lower baseline production of HIF-1α (consistent with our protein expression results). In this same group, both HIF-3α (which may lead to decreased basal HIF-1α mRNA expression [49]) and VHL (which destroys ubiquitinated HIF-1α [50]) mRNA were over-expressed, both consistent with the lack of HIF-1α expression seen on Western blotting.

### Limitations

Our study has several limitations. It was conducted in rats, which limits its applicability in humans. Because accurate, continuous measurement of Cyt_OX_ requires complete immobility which could only be achieved under general anesthesia, our animal model only included approximately 8 h of exposure to inflammation (the concentration of bacteria in blood peak at approximately 6–12 h after cecal ligation [26, 27], but ATP depletion does not occur until later), and did not include a group with no exposure to any anesthetic agents. Thus, our results cannot be generalized to non-anesthetized animals or late stage inflammation and sepsis. Because the propofol group received intraperitoneal ketamine-xylazine for induction (anticipated to last 20–30 min), and not a pure propofol anesthetic, we cannot exclude potential confounding effects from these two induction agents. mRNA data, while useful for hypothesis generation, requires confirmation with inhibitory studies in order to confirm any mechanistic hypotheses.

### Conclusions

Isoflurane and propofol lead to differential metabolic responses to inflammation. Isoflurane leads to development of lactic acidosis and reduction in Cyt_OX_ as compared to propofol. In the setting of inflammation, isoflurane led to increased oxidative stress, HIF-1α, iNOS, bcl-2, and HO-1 overexpression. HIF-1α expression in the setting of inflammation and isoflurane is likely mediated by both increased oxidative stress as well as increased Hsp90. In contrast, propofol attenuates inflammation-induced oxidative stress in the brain, preventing cerebral HIF-1α expression. Propofol also facilitates inflammation-induced expression of NF-κβ inhibitor mRNA, which may prevent transcription of basal HIF-1α. Many of these findings (increased oxidative stress, HIF-1α and bcl-2 expression after isoflurane exposure) are consistent with prior work on APC and may play a role in the organ protection observed by previous authors during sepsis [3–7]. Future work is needed to better understand the potential protective role of heat shock proteins and the adenosine 2A receptor in septic animals exposed to VAAs, as well as to translate these findings into improvements in relevant clinical outcomes in humans.

## Materials and methods

This study was approved by the animal care and use committee (IACUC) at the University of Virginia (protocol 4140) and conforms with the *Guide for the Care and Use of Laboratory Animals, 8th Edition* published by the National Institutes of Health (revised 2011). In order to allow other investigators to accurately assess the reproducibility of our work, we have included a more detailed explanation of our methods in the Additional file 3: Digital Content: Digital Content.

### General preparation

Ten-week-old male Sprague–Dawley rats (Envigo Corporation, Huntingdon, Cambridgeshire, UK) weighing on average 341 g were induced with either 5% isoflurane in 100% oxygen for 12 min (isoflurane group) or 100–10 mg/kg ketamine-xylazine (propofol group) injected intraperitoneally, after which they were orotracheally intubated with a 16 gauge intravenous catheter and connected to a mechanical ventilator (initial settings 1 Hz, 3.2 mL tidal volume, FiO_2_ 1.0). Minute ventilation was adjusted to keep end tidal CO_2_ (ETCO_2_) between 35 and 45 mm Hg. The right femoral artery (cut-down) and either a tail vein or the right femoral vein were cannulated with 24 gauge intravenous catheters. General anesthesia was maintained with either isoflurane (starting dose 1.5%, inspired) in 100% oxygen or propofol (average infusion rate 367 µg/kg/min) and depth of anesthesia was confirmed by toe pinching every 30 min. Temperature was measured using a rectal probe and controlled with a heating pad to a target of 37 °C. Arterial oxygen saturation (SpO_2_) was measured on the left lower extremity food pad.

### Near infrared spectroscopy

To minimize the interference of the scalp with our spectroscopy readings, we exposed the parietal bones of the skull bilaterally by dissecting the temporalis muscles through scalp incisions. Using a stereotactic frame we positioned emission and receiving fiberoptic bundles on opposite sides of the skull, on-axis with one another. The emission fiber was connected to a halogen light source and the receiving fiber was connected to a spectrometer (QE-Pro, Ocean Optics, Dunedin, FL), the light source output was recorded using a second spectrometer, and a transfer function was used to correct for minor differences between the two. A differential pathlength factor (DPF) correction was applied to the tissue spectra as previously described [19]. Both waveforms were smoothed over 20 nm, after which a broadband near infrared spectroscopy algorithm (750–925 nm, 1 nm intervals) was utilized to measure changes in the relative concentration of hemoglobin, oxyhemoglobin, and Cyt_OX_ [19].

### Surgical procedure(s) and sample harvesting

After initiation of general anesthesia and achievement of both arterial and venous access, 2 mL of arterial blood was withdrawn for analysis and replaced with 2 mL of warm normal saline (NS). Among the blood samples, 200 μL was utilized for arterial blood gas (ABG) analysis (CG4^+^ and Chem8^+^ cartridges, Abbot i-Stat, Lake Bluff, IL) and the remainder was injected into a serum collection tube for 45 min, after which it was centrifuged at 4 °C, the serum separated, and stored at − 80 °C.

The abdomen was shaved and prepared with three swipes of 70% isopropyl alcohol swabs, followed by scrubbing with 3 mL of 2% chlorhexidine gluconate and 70% isopropyl alcohol. A sterile drape was applied over the entire surgical field. Using sterile technique, a 2–3 cm midline celiotomy was performed. Animals were divided into sham (S) and cecal ligation and puncture (CLP) groups in alternating fashion, first for isoflurane (24 total) and then for propofol-based (24 total) anesthetics. In the controls, the cecum was identified, removed from the abdomen, and replaced back into the abdomen. In the animals exposed to inflammation, the cecum was identified and ligated (~ 90% by volume) with a size 0 silk suture, then 10 holes were placed using a 16 gauge needle (5 on the mesenteric border, 5 on the anti-mesenteric border), and patency was confirmed by extruding fecal contents through each hole.

In all groups, the celiotomy was closed in two layers—first, the muscular layer with 4–0 running silk sutures, followed by skin closure with clips. The animal was then placed in the prone position, the skull was trans-illuminated with a halogen light source, and commencement of NIRS recordings began. Hypotension, defined as mean arterial pressure < 80 mm Hg, was initially treated with intravenous warm NS (up to 3.3 mL/kg/h). If this was not effective, the anesthetic dose was reduced (and depth of anesthesia confirmed) when possible.

All animals were maintained under general anesthesia for 8 h after the celiotomy, after which an additional 2 mL of arterial blood was drawn for analysis. The stereotactic frame was removed, depth of general anesthesia was confirmed, bilateral burr holes were placed, and animals were euthanized using the freeze blow technique, in which the supratentorial brain was ejected onto a liquid N_2_-cooled copper plate using a high pressure air hose, immediately submerged in liquid N_2_, and stored at − 80 °C until used for biochemical analysis.

Immediately prior to analysis, the extracted tissue was removed from storage at − 80 °C and individually finely ground in liquid N_2_ with a pestle and mortar. Ten to fifty milligrams of tissue powder from each sample were used to measure ADP:ATP ratio, protein expression, and oxidative stress.

### Enzyme-linked immunosorbent assays (ELISA)

Arterial blood was immediately placed in BD Vacutainer tubes (Becton Dickson, Franklin Lakes, NJ) after collection, mixed by hand, and kept at room temperature for 45 min, after which it was centrifuged (2000 g) at 4 °C for 20 min. Using a micropipetter, the serum was removed and immediately stored at − 80 °C for analysis. Interleukin (IL)-6 (RAB0311, Sigma, St. Louis, MO), IL-1β (RAB0277, Sigma), and tumor necrosis factor (TNF)-α (ab100785, Abcam, Cambridge, UK) were measured according to the manufacturer’s instructions. For all assays, both the standards and unknowns were measured in duplicate.

### ADP:ATP ratio

The ratio of ADP to ATP was measured using bioluminescence kit (MAK135, Sigma) according to the manufacturer’s instructions. Briefly, a working reagent was added to the pulverized cells, inducing lysis and releasing both ADP and ATP. The addition of luciferase and d-Luciferin to the lysed product allowed for the measurement of ATP using a luminometer to measure relative light units (RLU_A_). ADP enzyme was then added to convert ADP to ATP, and an additional luminescent measurement (RLU_C_) combined with a measurement of background luminescence (RLU_B_) resulted in an estimate of ADP:ATP ratio based on the following equation:

$${\text{ADP:ATP}} = {\text{RLU}}_{\text{A}} /\left( {{\text{RLU}}_{\text{C}} - {\text{ RLU}}_{\text{B}} } \right)$$

### Immunoblotting

Fifty milligrams of tissue powder was suspended in 500 μL of ice cold phosphate buffer saline (PBS) containing a commercially-prepared protease and phosphatase inhibitor cocktail (Halt™, Thermo-Fisher #78443, Waltham, MA). Samples were gently mixed, centrifuged at 10,000 rpm for 5 min at 4 °C, and the pellet was collected and used either for nuclear protein extraction or for total protein lysis preparation.

Nuclear proteins were extracted using commercially available mammalian cell and tissue nuclear extraction kit (abcam ab113474, Cambridge, MA) by following the manufacturer’s protocol with the exception of adding 100 uM CoCl_2_ to the extraction buffer (RIPA) in order to stabilize prolyl hydroxylase and thereby preventing HIF-1α degradation in the presence of atmospheric oxygen.

For total protein extraction, 250 μL of ice cold lysis buffer (RIPA) containing a protease and phosphatase inhibitor cocktail (Halt™) was added to the pellet, which was subsequently sonicated on ice (3 cycles of 10 pulses, power 5, Fisher Scientific Sonic Dismembrator Model F60, Pittsburgh, PA). Protein lysates were centrifuged at 13,000 rpm for 20 min at 4 °C.

Protein concentrations were determined using a Bicinchoninic Acid (BCA) kit (Thermo Scientific). Ten to thirty micrograms of total protein (50 ug for HIF-1α) was heat-denatured at 95C, loaded onto 4–20% Tris–Glycine polyacrylamide gradient gels (Bio-Rad, Hercules, CA), and electrophoresed. Proteins were transferred to PVDF membrane (Millipore, Darmstadt, Germany), blocked at room temperature for 1 h in SuperBlock phosphate-buffered saline (PBS) Blocking Buffer (Thermo-Fisher # 37515) and incubated at 4 °C overnight with primary antibodies: anti-HIF-1α (1:2000, rabbit-mAb # 14179, Cell Signaling [Danvers, MA]; note: for HIF-1α, overnight incubation with primary antibodies was repeated after washing three times with PBST), anti-heme-oxygenase-1 (HO-1, 1:3000, mouse-mAb # NBP1-97507, NovusBio [Littleton, CO]), or anti-B cell lymphoma 2 (bcl2, 1:5000, mouse-mAb # NB100-78543, NovusBio), anti-inducible nitric oxide synthase (iNOS, 1:1500, rabbit-mAb # 13120, Cell signaling). For loading controls, anti-β-tubulin (1:2000, mouse-mAb # NB 120-7792, NovusBio) and anti-Lamin B1 (1:2000, rabbit-mAb # 13435, Cell Signaling) were used.

After primary antibody incubation, membranes were washed three times in PBST for 10 min. Membranes were then incubated with horseradish peroxidase–conjugated secondary antibodies (1:1000–15,000, depending on the primary antibody used, Santa Cruz Biotechnology [Dallas, TX] and Cell Signaling], see Additional file 3: Digital Content: for more details) for 1 h at room temperature. Immunoreactivity was detected using enhanced chemiluminescence substrate (Super Signal West Femto; Thermo Scientific). Images were captured using GBOX (Chemi XR5; Syngene), and gels were analyzed densitometrically using the computerized image analysis software (Gene Tools from Syngene). Target protein bands were normalized to loading controls β-tubulin or Lamin B1.

### Measures of oxidative stress

For measurement of protein carbonyls, tissue powder was suspended in ice cold PBS + 0.5 mM EDTA buffer, mixed, and centrifuged at 4 °C. The pellet was suspended in ice cold PBS + 0.5 mM EDTA buffer containing a protease and phosphatase inhibitor cocktail (Halt™), followed by sonication on ice. Samples were centrifuged and 10% streptozocin solution was added to the supernatant and incubated at room temperature to remove nucleic acids. Samples were then re-centrifuged, the supernatant was collected and used for a commercial protein carbonyl content assay. Five microliters of the sample were set aside for measurement of protein concentration using the BCA assay.

Protein carbonyl content was measured using the Protein Carbonyl Content Assay Kit (MAK094, Sigma-Aldrich, St. Louis, MO) in accordance with the manufacturer’s instructions. This assay is based on the 2,4-dinitrophenylhydrazine (DNPH) reaction. DNPH was added to each sample which was then vortexed and incubated at room temperature. TCA was added followed by vortexing, incubation on ice, centrifugation at − 4 °C, and removal of supernatant. Acetone was added to the pellet which was sonicated and centrifuged. Guanidine solution was added and sonication repeated, after which absorbance was measured at 375 nm (A_375_). Carbonyl content was calculated based on A_375_ and indexed to protein content.

Brain tissue *S*-nitrosylation was analyzed using a *S*-nitrosylation Western blot kit (Thermo Scientific # 90105) according to the manufacturer’s instructions. Ground tissue powder was lysed in HENS buffer (100 mM HEPES, pH 7.8; 1 mM EDTA; 0.1 mM Neocuproine; 1% SDS) followed by sonication and centrifugation. The supernatant was collected, and protein concentrations were determined using BCA kit (Thermo Scientific).

Two microliters of 1 M methyl methanethiosulfonate (MMTS) was added to the protein sample, the sample was vortexed and incubated at room temperature. To precipitate the protein, pre-chilled acetone was added to the samples and incubated at − 20 °C. Samples were centrifuged and the pellet was air dried, then re-suspended in HENS buffer. Iodo TMT labeling reagent and sodium ascorbate were added, then vortexed and incubated in a cold room overnight. Pre-chilled acetone was added to the samples which were incubated at − 20 °C, re-centrifuged at 10,000 rpm for 15 min, after which the pellet was air dried and re-suspended in HENS buffer.

To this iodo TMT-labelled sample (50 μL) 12.5 μL of 4× reducing Laemmli sample buffer was added and heated for 5 min at 99 °C. Twenty microliters of each sample was loaded onto 4–20% TGX gels. Proteins were transferred to PVDF membrane, blocked at room temperature for 1 h in SuperBlock Blocking Buffer (Thermo-Fisher # 37515) and incubated at 4 °C overnight with anti-TMT antibody (1:2000, Thermo-Fisher # 90075). Membranes were washed three times in TBST for 10 min then incubated with anti-mouse IgG-HRP conjugated (1:15,000) secondary antibody for 1 h at room temperature. Immunoreactivity was detected using enhanced chemiluminescence substrate (Super Signal West Femto; Thermo Scientific). Images were captured using GBOX (Chemi XR5; Syngene), and gels were analyzed densitometrically using the computerized image analysis software (Gene Tools from Syngene). Loading controls (total protein) were stained with Ponceau S solution. *S*-nitrosylation was indexed to total protein.

### RNA expression

In order to identify differences between anesthetic agents in the setting of inflammation that were not part of our primary analysis, we compared mRNA expression in both control and CLP rats exposed to both isoflurane and propofol. Results were screened by the authors for genes related to metabolism, oxidative phosphorylation, oxidative stress, and cellular protection (see Table 2).

### RNA isolation and library preparation

Tissue pooling was performed as in Table 3. Total RNA from pooled rat brain samples was isolated using the RNeasy plus mini kit (Qiagen) and concentrations were estimated using NanoDrop (ThermoFisher Scientific, we used UVA Biorepository and Tissue Research Facility for RNA extraction). 200 ng of total RNA from each sample was submitted to University of Virginia Genome Analysis Technology Core for mRNA Seq using Illumina Next-Generation sequencing.

**Table 3:** Grouping of tissue samples utilized in RNA sequencing

Treatment group	Number of samples	Pooling per each sample	Total number of rats used
Propofol: control/sham	4	3	12 (4 × 3)
Propofol: sepsis/CLP	4	3	12 (4 × 3)
Isoflurane: control/sham	4	3	12 (4 × 3)
Isoflurane: sepsis/CLP	4	3	12 (4 × 3)

Total RNA was quantified using the Invitrogen Qubit 3 Fluorometer (ThermoFisher Scientific) and quality was assessed on the Agilent Technologies 4200 TapeStation. Samples with RNA integrity numbers of 7.7 and above were used to prepare libraries using the NEBNext Ultra II Directional RNA Library Prep Kit for Illumina. All RNAs were processed at the same time using master mixes and validated standard operating procedures established by the UVA School of Medicine’s Genome Analysis and Technology Core. Briefly, 1 µg of total RNA was used to isolate mRNA using NEBNext Poly(A) mRNA Magnetic Isolation Module, followed by fragmentation and first and second-strand cDNA synthesis and fragmentation as recommended by the manufacturer. The resulting cDNA was end-repaired and then adenylated, in preparation for the ligation of sequencing adapters. Unique barcodes were added to the ligation products via PCR, and the final purified libraries were quantified and sized using the Invitrogen Qubit 3 Fluorometer and Agilent TapeStation.

#### Next-generation sequencing and quality control

Individually barcoded libraries were adjusted to equal molar concentration and pooled (16 per run) to a 4 nM solution, which was then denatured in 0.1 sodium hydroxide, following the Illumina recommended procedure. Before each run, the denatured pool was diluted to 1.3 pM before loading on the Illumina NextSeq 75 bp High Output sequencing kit reagent cartridge. Both pools were run sequentially on the Illumina NextSeq 500 for single-end sequencing. After transfer to the Illumina Base Space interface, the quality of the runs was assessed by the numbers of reads in millions passing filter (540 M and 505 M, respectively) and the % of indexed reads (97.5%).

#### RNA expression analysis

Read alignments and expression read counts were performed using STAR [51]. The reference genome of *Rattus norvegicus* (Rat) version rn5 from UCSC was used for read assembly and mapping. The resulting read counts were summarized per gene and was imported into R for performing differential expression of genes using DESeq2 [52]. We had 4 biological replicates, each for the CLP and the control group of rats in this study (though technically each one of these were pooled from 3 samples). We analyzed the treatment (sepsis) effect on the expression of genes under each anesthetic background. We performed FDR (False Discovery Rate [Benjamini–Hochberg]) correction of *p* values obtained for each gene in the study and identified significant genes using a FDR/Padj threshold of less than 0.05. A list of genes was tabulated that were significantly up- or down regulated in the CLP group of rats (compared to control/sham) for each anesthetic (propofol and isoflurane).

### Power analysis and statistical comparisons

The primary outcome of this study was the change in Cyt_OX_ 8 h after celiotomy. We hypothesized that animals subjected to inflammation would experience significant reductions in Cyt_OX_ in comparison with controls. Based on pilot data from our laboratory we expected Cyt_OX_ to decrease by 0.014 arbitrary units (s = 0.0083) after exposure to inflammation, suggesting an effect size of approximately 0.6 and that two groups of 12 animals would be needed to reliably discriminate between groups (one-way ANOVA, α = 0.05, 1 − β = 0.80). Because we are interested in the impact of inflammation on subcellular energetics using both VAA and GABA agonists as anesthetic agents, we require four total groups of 12 animals (48 total).

All data (Cyt_OX_, protein expression, ADP:ATP ratio, measures of oxidative stress, and laboratory values) were tested for normality using the D’Agostino–Pearson normality test. Groups of normally distributed data (control vs. inflammation) were compared using the t-test (parametric) or Mann–Whitney test (non-parametric) as appropriate. When paired data were available (e.g. interleukins), paired tests were utilized to account for interindividual variability. All calculations were performed in GraphPad Prism 7 (GraphPad Software, La Jolla, CA) and MATLAB (Mathworks, Natick, MA).

Unless otherwise noted, parametric grouped data are presented as mean and 95% confidence intervals, whereas non-parametric grouped data are presented as median and interquartile range. Time series NIRS data were analyzed using a permutation F-test, performed by a biostatistician (TM).

## Additional files

**Additional file 1.** Statistical Analysis of NIRS Data: detailed statistical analysis of NIRS data produced by biostatistician Dr. Timothy McMurry.

**Additional file 2. Table S1: ** All statistically significant up and down-regulated genes identified by RNA sequencing.

**Additional file 3**. Supplemental Digital Content: more detailed explanation of our methods with particular emphasis on HIF-1α Western blotting.

## Data Availability

Every effort to include relevant data in the manuscript or supplemental appendix has been made. Any data not included in the manuscript or in the supplemental appendix will be available at Open Science Framework (https://osf.io) under the Project Title “Brain Subcellular Energetics (Rat) After Cecal Ligation and Puncture” as well as via email from either of the corresponding authors.

## Abbreviations

APC: anesthetic preconditioning
bcl-2: B-cell lymphoma 2
BCA: bicinchoninic acid
CLP: cecal ligation and puncture
CSF: cerebrospinal fluid
DNPH: 2,4-dinitrophenylhydrazine
DPF: differential pathlength factor
ETC: electron transport chain
ETCO2: end tidal CO_2_
hsp: heat shock protein
HO-1: heme oxygenase 1
HIF-1: hypoxia-inducible factor 1
iNOS: inducible nitric oxide synthase
IGF: insulin-like growth factor
IL: Interleukin
IPC: ischemic preconditioning
LDH: lactate dehydrogenase
MMTS: methyl methanethiosulfonate
NIRS: near infrared spectroscopy
NS: normal saline
CytOX: oxidation state of cytochrome aa3
PBS: phosphate buffer saline
PHD: prolyl hydroxylases
ROS: reactive oxidative species
RLU: relative light units
S: sham
StO2: tissue oxygenation
TCA: tricyclic acid
TNF: tumor necrosis factor
VAAs: volatile anesthetic agents
