# Supplementary material for: Impact of inflammation on brain subcellular energetics in anesthetized rats
Source: BMC Neurosci. 2019 Jul 15;20:34. doi: 10.1186/s12868-019-0514-8 (PMC6631861; doi:10.1186/s12868-019-0514-8)
Supplement: Supplementary file 1 — Additional file 1. Statistical Analysis of NIRS Data: detailed statistical analysis of NIRS data produced by biostatistician Dr. Timothy McMurry. [file 12868_2019_514_MOESM1_ESM.pdf]

Analysis of the NIRS data set

Tim McMurry
tmcmurry@virginia.edu

July 25, 2018

I used a functional data approach to comparing the 3 outcome measures HgbR, HgbO, and CytoD across anesthesia (Isoflurane/Propofol) and Treatment (Control/Sepsis). The idea is similar to a standard ANOVA, except analyses are conducted in parallel across all times. A standard reference is Ramsay and Silverman (2005). We follow the approach outlined in Chapters 12 and 13 for fitting functional linear models and performing hypothesis tests via permutation tests.

The first step in this approach is to smooth the input data. An example is shown below with the original data in red, and the smoothed data overlaid in black.

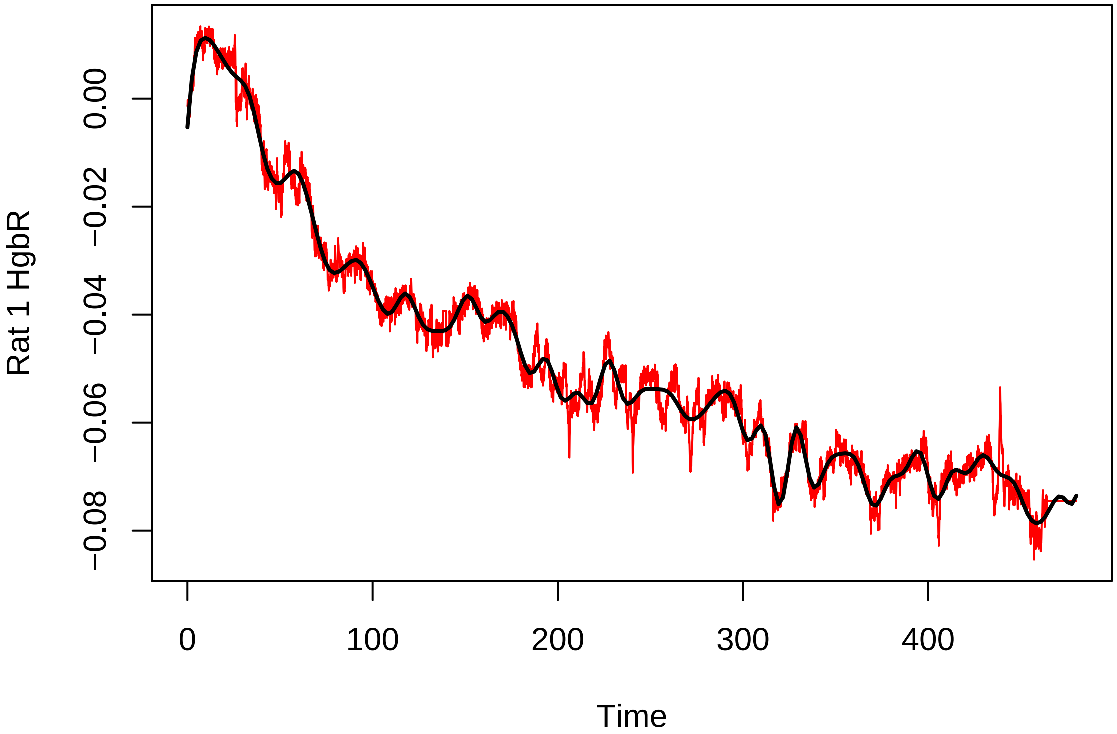

Since most rats had some missing data, I interpolated all missing data. If data was missing at the end of the time period, I extended the last observed value. The final data is shown below.

```
## [1] "done"
## [1] "done"
```

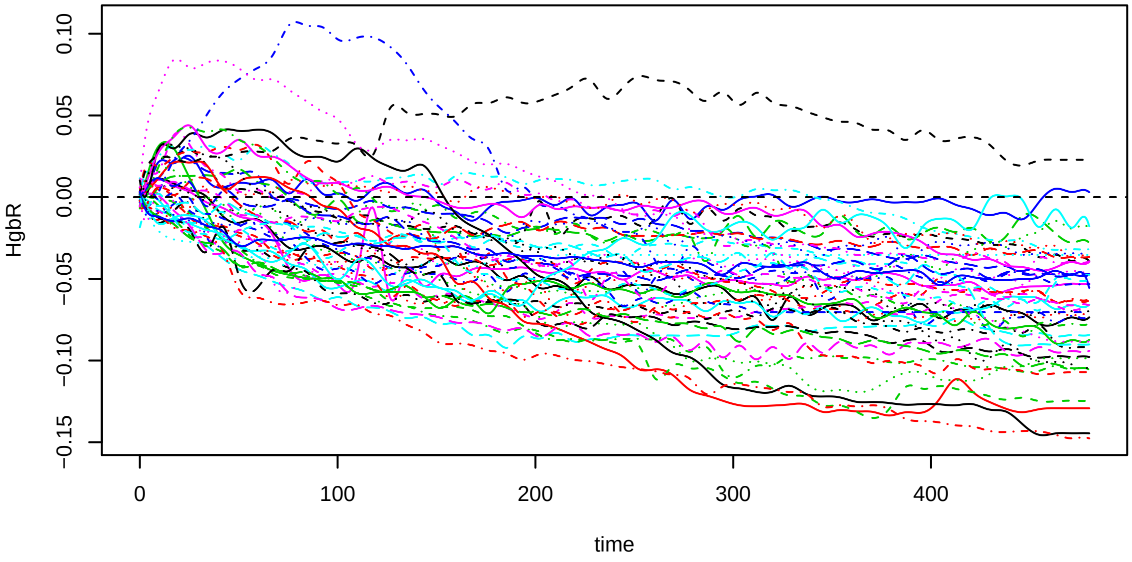

HgbR
0.10
0.05
0.00
−0.05
−0.10
−0.15
0
100
200
300
400
time

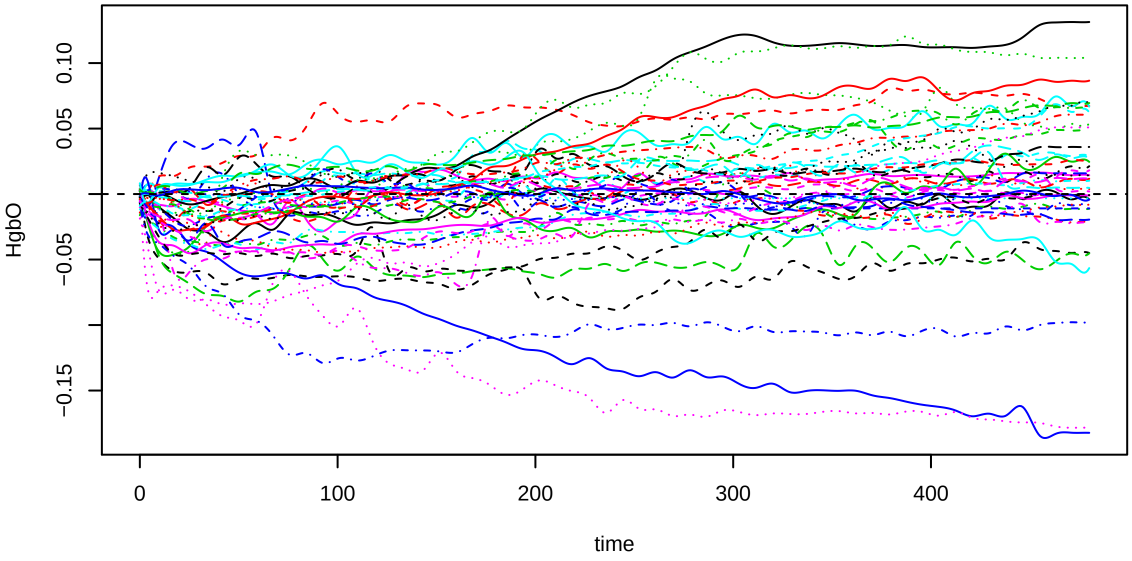

HgbO
0.10
0.05
−0.05
−0.15
0
100
200
300
400
time

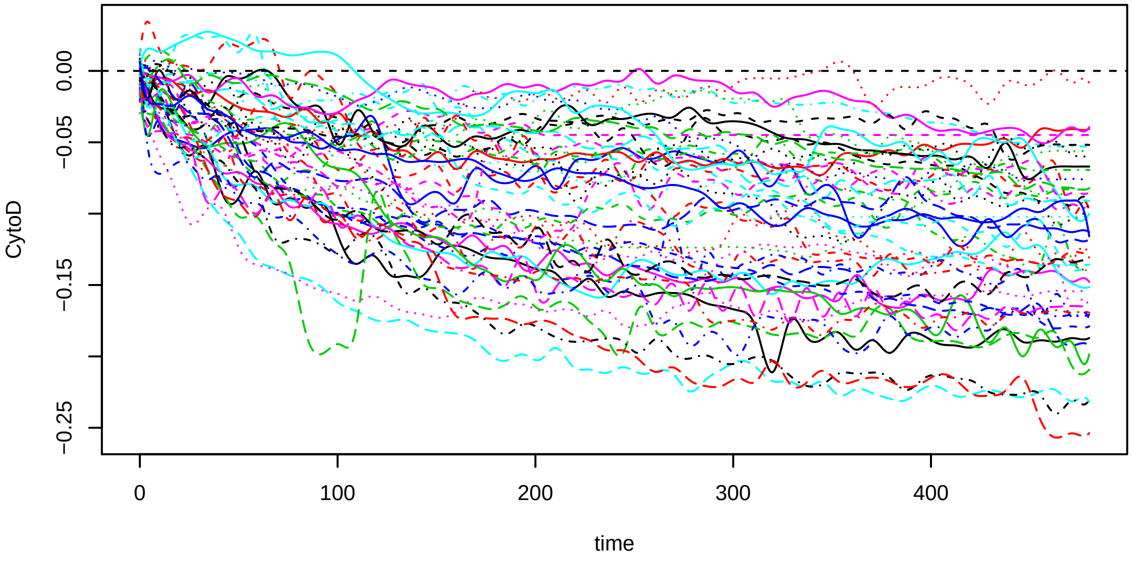

CytoD
0.00
−0.05
−0.15
−0.25
0
100
200
300
400
time

```
## [1] "done"
```

ANOVA Models

The fundamental approach is the permutation test. For example, to test whether HgbR is associated with Anesthesia and Treatment, we fit the the main model, where HgbR is predicted by Anesthesia and Treatment. Then to estimate a p-value, we look at the distribution of F-statistics when there is no effect; this is generated by repeatedly shuffling the order of the HgbR curves relative to the treatment assignments, and refitting the models to the shuffled data. We then compared F-statistics across time. The plots show the pointwise hypothesis tests at each time (small blue dots), and the global test for significance (blue dashes) which controls for comparing at all times.

Note that the F-values in the output below are not scaled correctly to compare with F-values tablulated in statistics books.

HgbR

Globally, HgbR does not show a statistically significant association with Anesthesia or Treatment:

```
## [1] "Estimated Computing time = 280 seconds."
```

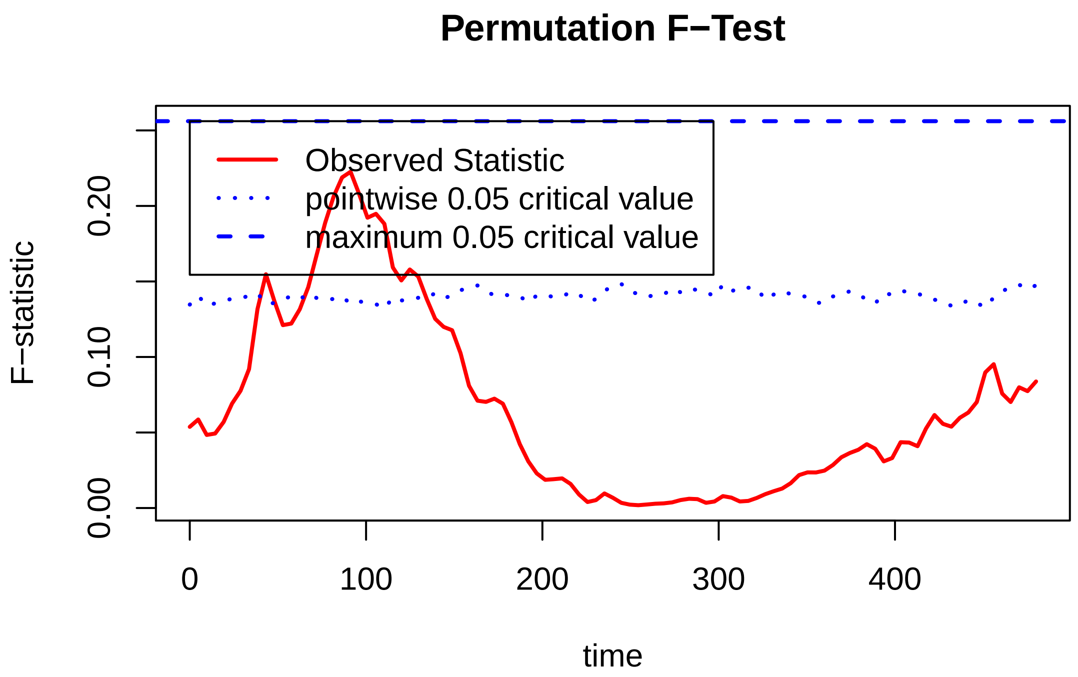

The p-value is 0.084.

HgbO

Globally, HgbO does not show a statistically significant association with Anesthesia or Treatment:

```
## [1] "Estimated Computing time = 470 seconds."
```

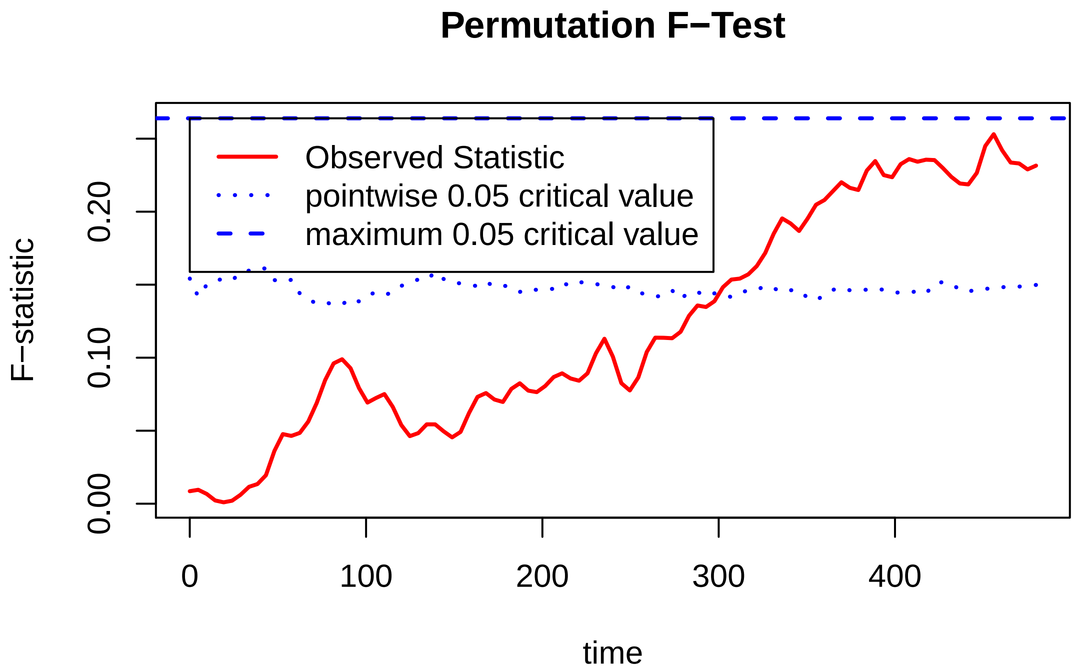

The p-value is 0.059.

CytoD

Globally, CytoD shows a statistically significant association with Anesthesia or Treatment:

```
## [1] "Estimated Computing time = 360 seconds."
```

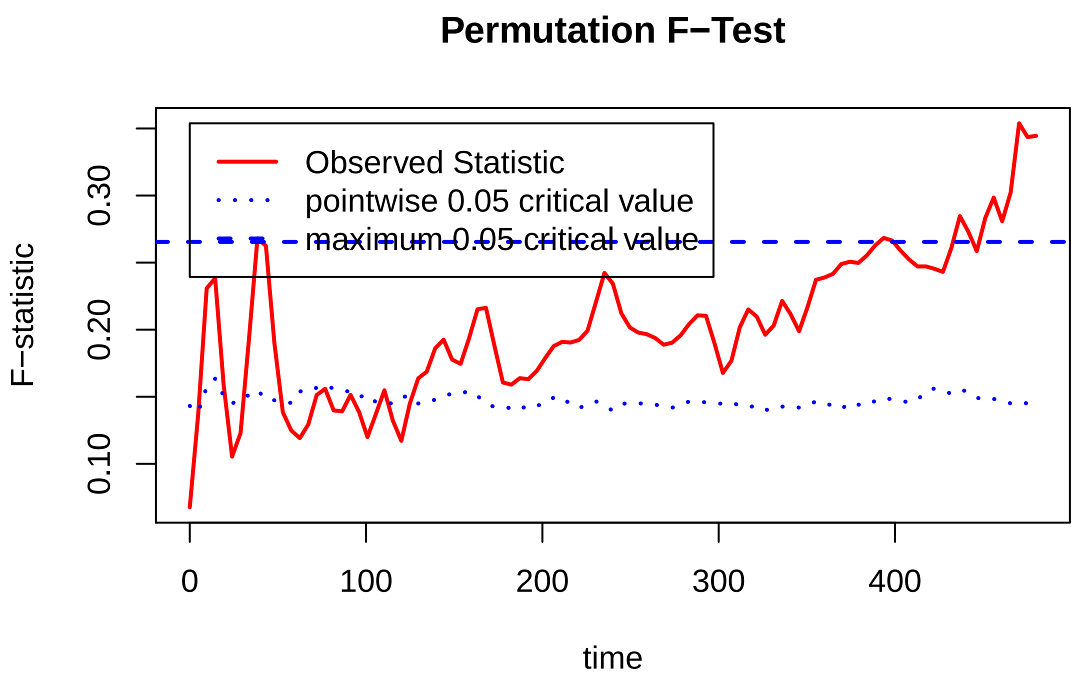

The p-value is 0.014.

Based on these findings, I then looked to see if the differences were do to Anesthesia or Treatment. The difference appears to be due to Anesthesia.

Evaluation of Treatment

```
## [1] "Estimated Computing time = 330 seconds."
```

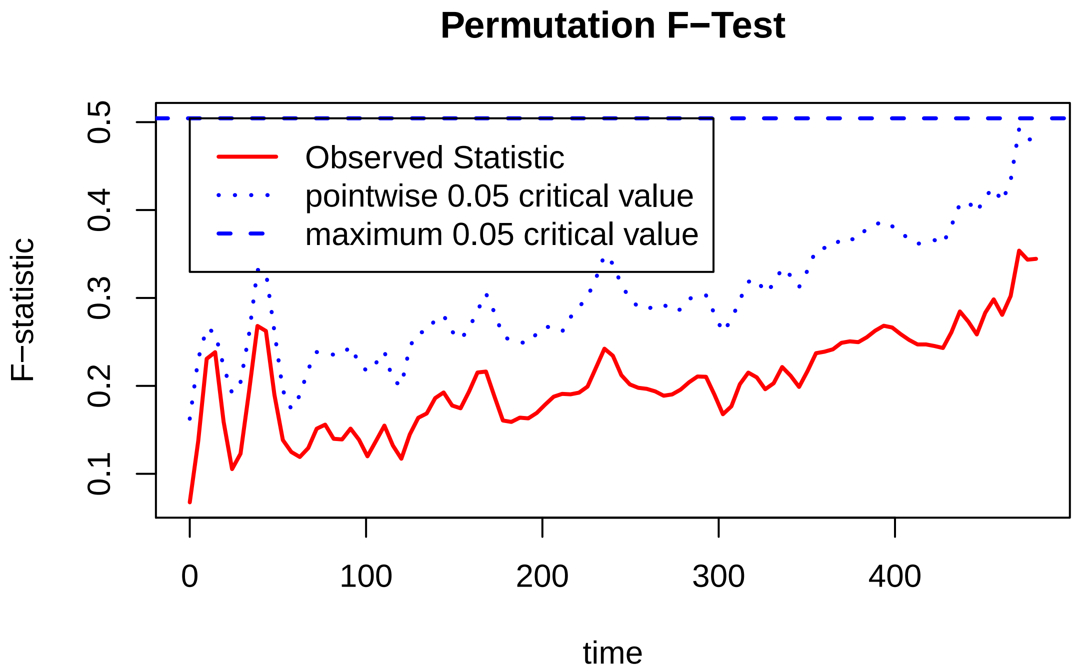

The p-value is 0.710.

Evaluation of Anesthesia

```
## [1] "Estimated Computing time = 410 seconds."
```

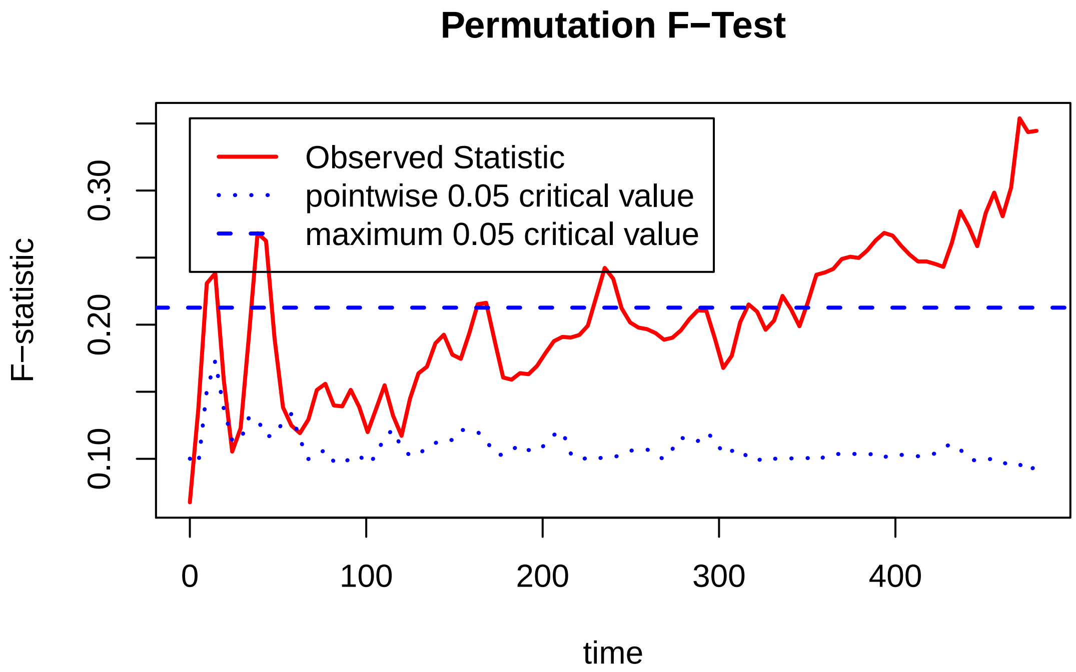

The p-value is 0.006. The estimated effect of Anesthesia in time is shown below.

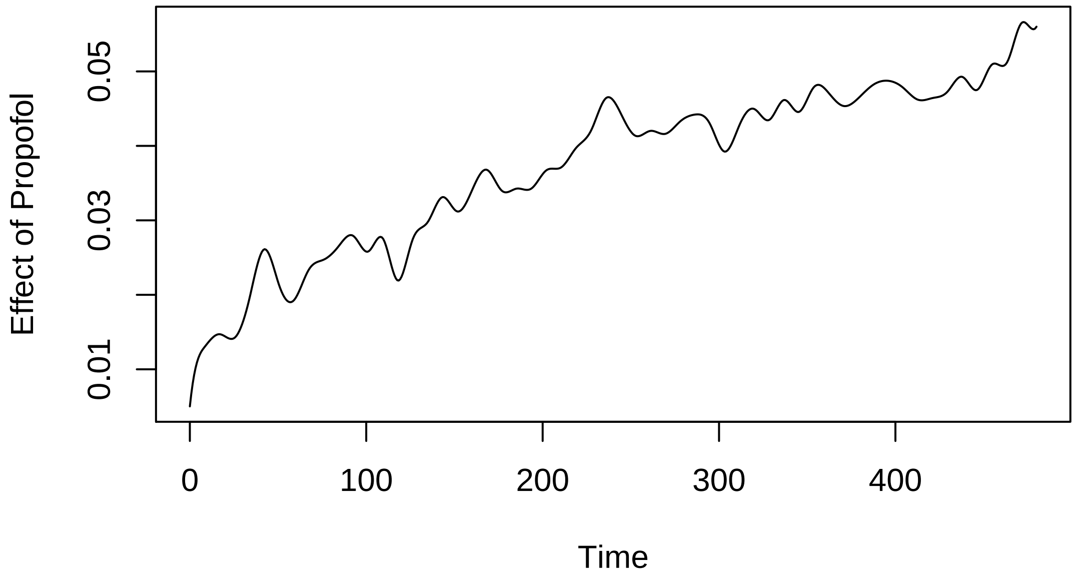


```
## [1] "done"
```

References

J.O. Ramsay and B.W. Silverman. *Functional Data Analysis*. Springer, New York, NY, 2nd edition, 2005.
