# Supplementary material for: Impact of inflammation on brain subcellular energetics in anesthetized rats
Source: BMC Neurosci. 2019 Jul 15;20:34. doi: 10.1186/s12868-019-0514-8 (PMC6631861; doi:10.1186/s12868-019-0514-8)
Supplement: Supplementary file 3 — Additional file 3. Supplemental Digital Content: more detailed explanation of our methods with particular emphasis on HIF-1α Western blotting. [file 12868_2019_514_MOESM3_ESM.docx]

**Additional file Digital Content**

**Methodology**

HIF-1 is a highly labile protein and is degraded by prolyl hydroxylase within minutes of exposure to atmospheric levels of oxygen.^1^ Thus, tissue handling can be a challenge. Much has been written about the difficulties associated with measuring HIF-1 expression and the purpose of this supplemental digital content is to describe our methodology (for both measurement of protein expression as well as oxidative stress) in a great level of detail than what might typically appear in a printed manuscript, so that other investigators can independently verify our work.

**Tissue Preparation**

Immediately prior to analysis, the extracted tissue was removed from storage at -80C and individually finely ground in liquid N_2_ with a pestle and mortar. During this process we constantly poured liquid nitrogen on the sample to ensure that it remained below -80C. Ten to fifty milligrams of brain or liver tissue powder from each sample were used to measure ATP:ADP ratio, protein expression, and oxidative stress. We found that grinding tissue into powder using liquid nitrogen N_2_ with a pestle and mortar optimized conditions for subsequent steps (e.g. nuclear extraction) without risking exposure to oxygen and biological activity that might alter our results (e.g. HIF-1 degradation).

**Immunoblotting**

Overall Approach (HIF-1):

1. Nuclear extract vs. whole cell lysate: HIF-1 is expressed in the nucleus and thus, in theory, use of nuclear extracts would increase the relative concentration of HIF-1 (or any nuclear protein) loaded into a SDS-PAGE gel for separation. On the other hand, because HIF-1 is so labile, any additional step that is not performed in a hypoxic chamber can theoretically increase the risk of HIF-1 degradation. We were able to manage this risk in two ways – first, by grinding the tissue using liquid nitrogen N_2_ with a pestle and mortar beforehand (see above), which facilitated the rapid performance of the nuclear extraction, and second, by adding CoCl_2_ to the extraction buffer in order to stabilize prolyl hydroxylase and thereby preventing HIF-1 degradation in the presence of atmospheric oxygen. CoCl_2_ has been shown to prevent HIF-1 degradation during Western blotting and, importantly, does not lead to HIF-1 expression in the absence of hypoxia.^2^


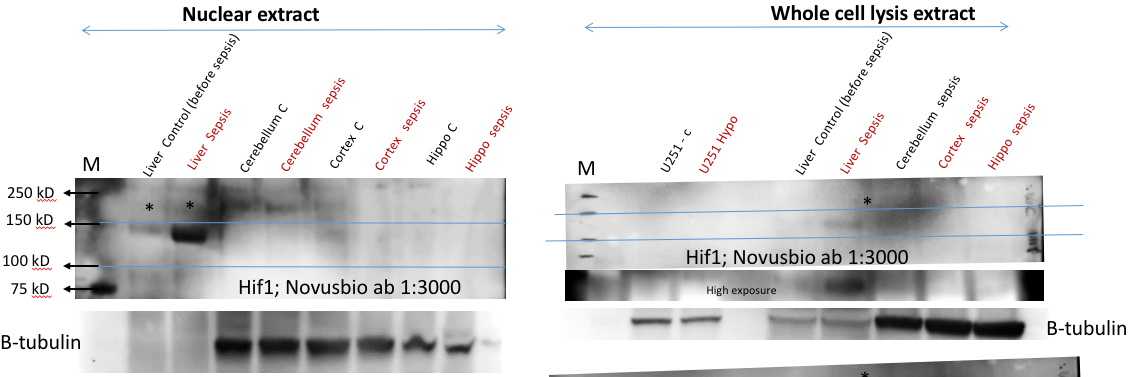


**Figure 1S**: demonstration that nuclear extract produces higher expression of HIF-1 than extract

from whole cell lysis

1. Antibody selection: we tested multiple antibodies from Cell Signaling and Novus Biologicals. The antibodies which gave us the strongest, most reliable and repeatable signals are reported in the table below.
2. Use of CoCl_2_: CoCl_2_ has been shown to prevent HIF-1 degradation during Western blotting and, importantly, does not lead to HIF-1 expression in the absence of hypoxia.^2^ We added it to the extraction buffer during our nuclear extraction step, as this was an opportunity for our tissue to be exposed to atmospheric oxygen while not at -80C.


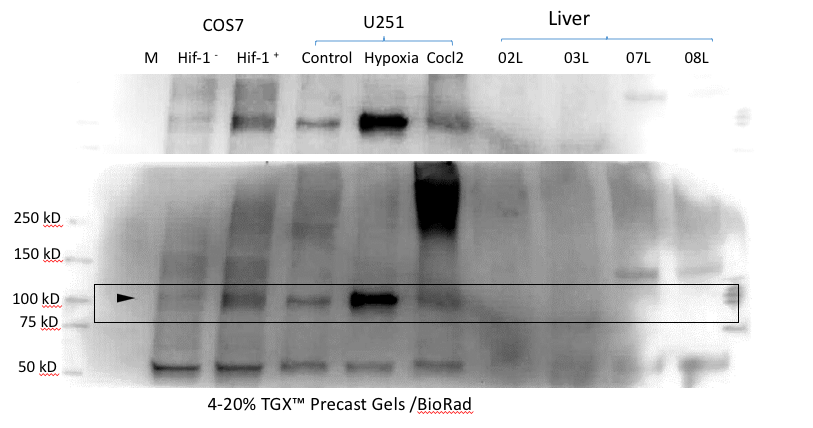


**Figure 2S**: demonstration that addition of CoCl_2_ to U251 cells does not lead to HIF-1 expression (rather, it prevents degradation)


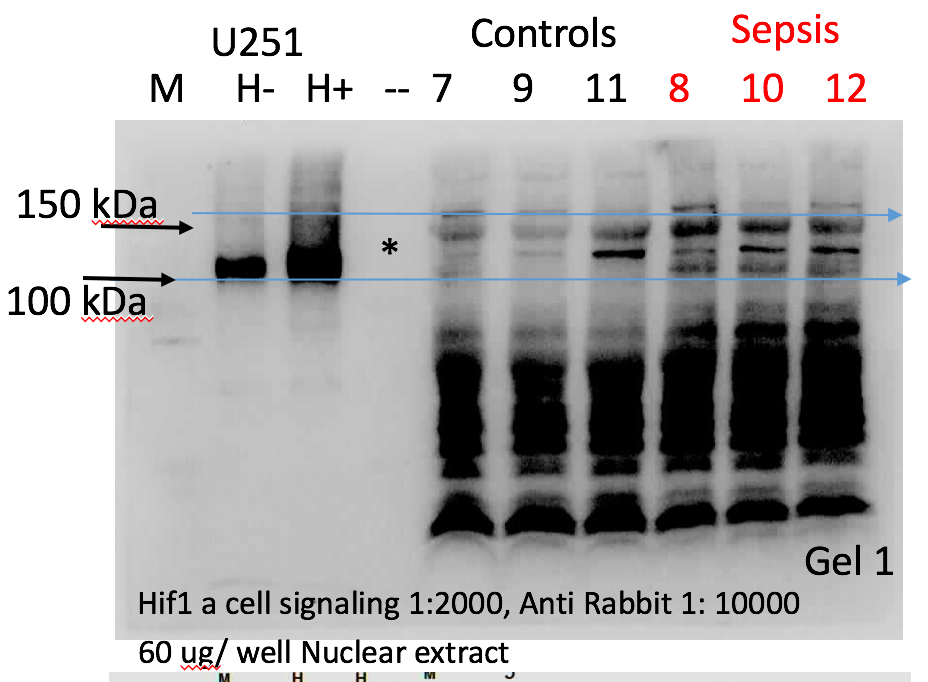


**Figure 3S**: demonstration of HIF-1 expression in Hypoxic U251 cells (Uh) as well as liver of control rats (7, 9, 11) and rats exposed to sepsis (8, 10, 12). Of note, HIF-1 expression due to hypoxia is significantly more than HIF-1 over-expression due to sepsis/inflammation

1. Amount of protein: there is some controversy about how much protein should be loaded into a SDS-PAGE gel for analysis of HIF. Some investigators suggest loading as much as 100 mg of protein. We found that this was excessive. While HIF-1 is not present in particularly high concentrations, even after nuclear extraction (at least when triggered by sepsis/inflammation; during hypoxia, when HIF-1 expression), use of too much total protein can potentially clog the pores of the gradient gel we used, and impede separation. Rather, we found that use of 50 mg total protein and particular attention to long exposure and repeated washing with the primary antibodies (see below) could effectively identify HIF-1 in our samples.
2. Confirmation with hypoxic cells: to ensure that we were measuring HIF-1, we purchased extract from COS7 cells exposed to hypoxia, and also exposed our own U251 cells to hypoxia and performed Western blotting using the same methodology we used in our tissue samples.


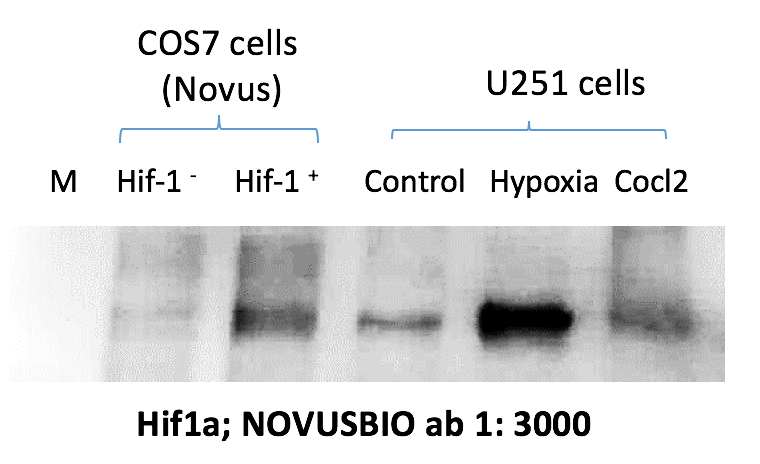


**Figure 4S**: demonstration of HIF-1 expression in extract from COS7 cells exposed to normoxia and hypoxia (purchased from Novus Biologicals) as well as control and U251 cells, U251 cells exposed to hypoxia, and U251 cells exposed to CoCl_2_


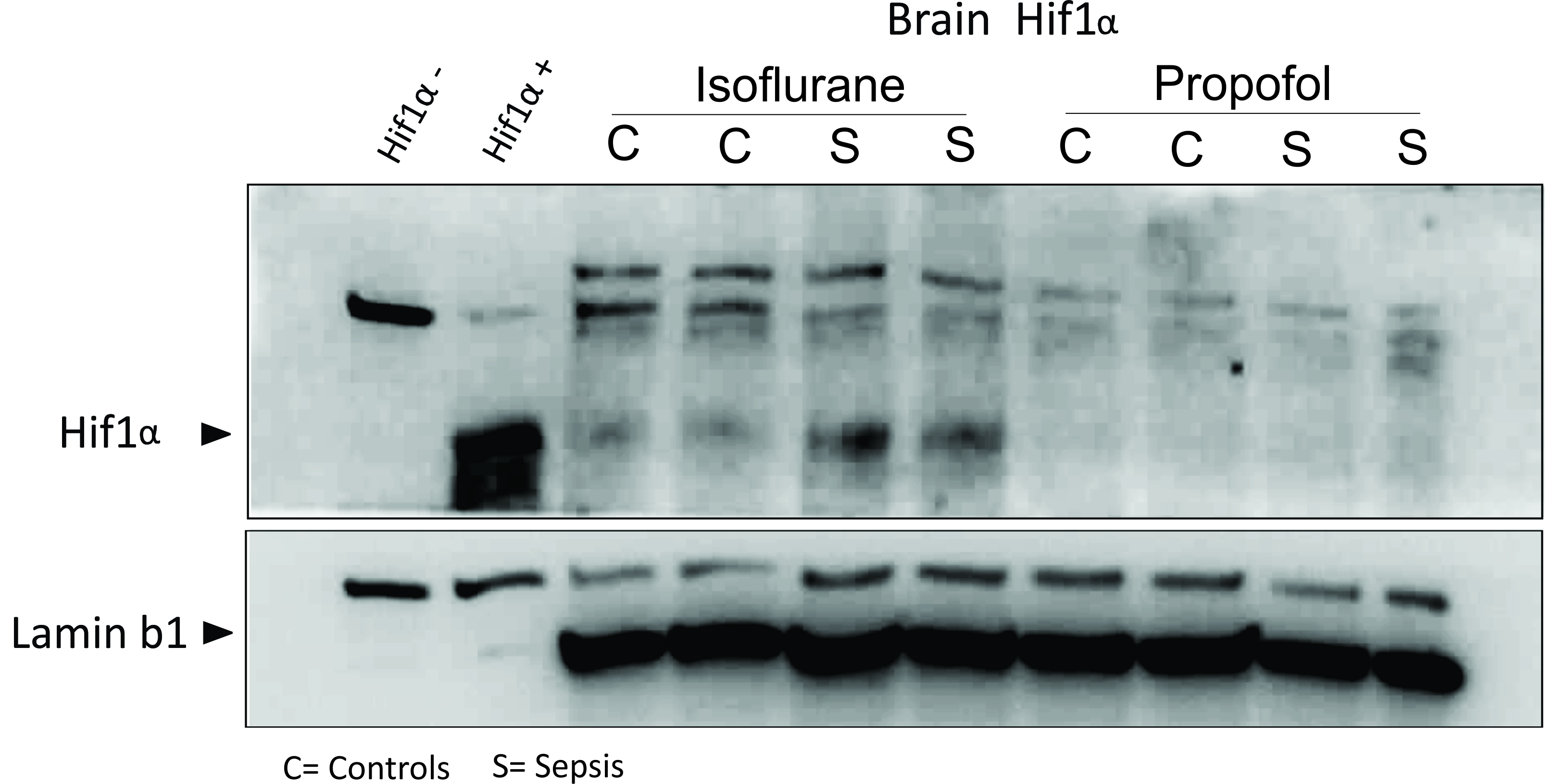


**Figure 5S**: demonstration of HIF-1 expression in tissue exposed to isoflurane but not propofol. Columns 1-2 are H9c2 cells under normaoxia and hypoxia (1% O_2_); rows 3-4 are isoflurane controls; rows 5-6 are isoflurane sepsis; rows 7-8 are propofol controls; rows 9-10 are propofol sepsis. You can clearly see increased HIF-1a expression in rows 5-6 as compared to rows 3-4, and also a complete lack of HIF-1a expression in rows 7-10. This confirms that our lack of HIF-1a expression in the propofol group is not due to methodological error(s).

Specific Methodology (all proteins):

50 mg of brain or liver tissue powder was suspended in 500 ul of ice cold PBS containing a commercially-prepared protease and phosphatase inhibitor cocktail (Halt^TM^, Thermo -Fisher #78443, Waltham, MA). Samples were gently mixed, centrifuged at 10,000 rpm for 5 minutes at 4^0^C, and the pellet was collected and used either for nuclear protein extraction or for total protein lysis preparation (this is a washing step to remove blood or other tissue contaminants)

Nuclear proteins were extracted using commercially available mammalian cell and tissue nuclear extraction kit (abcam ab113474, Cambridge, MA) by following the manufacturer’s protocol with the exception of adding 100 uM CoCl_2_ to the extraction buffer (we used RIPA for this stage of the nuclear extraction because its contents were more clearly described) in order to stabilize prolyl hydroxylase and thereby preventing HIF-1 degradation in the presence of atmospheric oxygen.^2^

For total protein extraction, 250 uL of ice cold lysis buffer (RIPA) containing a protease and phosphatase inhibitor cocktail (Halt^TM^) was added to the pellet, which was subsequently sonicated on ice (3 cycles of 10 pulses, power 5, Fisher Scientific Sonic Dismembrator Model F60, Pittsburgh, PA). Protein lysates were centrifuged at 13,000 rpm for 20 minutes at 4^0^C.

Protein concentrations were determined using BCA kit (Thermo Scientific). 10-30 μg of total protein (50 ug for HIF-1α) was heat-denatured at 95C, loaded onto 4 to 20% Tris-Glycine polyacrylamide gradient gels (Bio-Rad, Hercules, CA), and electrophoresed. Proteins were transferred to PVDF membrane (Millipore, Darmstadt, Germany), blocked at room temperature for 1 h in SuperBlock (PBS) Blocking Buffer (Thermo-Fisher # 37515) and incubated at 4°C overnight with primary antibodies; anti-HIF-1α (1:2000, rabbit –mAb # 14179, Cell Signaling [Danvers, MA]; note: for HIF-1α, overnight incubation with primary antibodies was repeated after washing three times with PBST), anti-HO-1 (1:3000, mouse- mAb # NBP1-97507, NovusBio [Littleton, CO]), anti-bcl2 (1:5000, mouse- mAb # NB100-78543, NovusBio), anti-iNOS (1:1500, rabbit-mAb # 13120, Cell signaling). For loading controls, anti-β-tubulin (1:2000, mouse-mAb # NB 120-7792, NovusBio) and anti-Lamin B1 (1:2000, rabbit-mAb # 13435, Cell Signaling) were used.

After primary antibody incubation, membranes were washed three times in PBST for 10 minutes. Membranes were then incubated with horseradish peroxidase–conjugated secondary antibodies (1:1000-15,000, depending on the primary antibody used, Santa Cruz Biotechnology [Dallas, TX] and Cell Signaling], for 1h at room temperature. Immunoreactivity was detected using enhanced chemiluminescence substrate (Super Signal West Femto; Thermo Scientific). Images were captured using GBOX (Chemi XR5; Syngene), and gels were analyzed densitometrically using the computerized image analysis software (Gene Tools from Syngene). Target protein bands were normalized to loading controls β-tubulin or Lamin B1.

|  | **HIF-1** | **Bcl-2** | **iNOS** | **HO-1** |
| --- | --- | --- | --- | --- |
| Location | Nuclear | Total Protein Lysate | Total Protein Lysate | Total Protein Lysate |
| Gel | 4-20% TGX 50ul capacity | 4-20% TGX 20-30 ul capacity | 4-20% TGX 20-30 ul capacity | 4-20% TGX 20-30 ul capacity |
| Protein loaded | 50 ug | 25 ug | 25 ug | 25 ug |
| Primary Antibody | rabbit –mAb # 14179  Cell signaling | mouse- mAb # NB100-78543  NovusBio | rabbit-mAb # 13120  Cell signaling | mouse- mAb # NBP1-97507  NovusBio |
| Primary Dilution | 1:2000 | 1:5000 | 1:1500 | 1:3000 |
| Secondary antibody | Rabbit | Mouse | Rabbit | Mouse |
| Secondary dilution | 1:15000 Santa Cruz | 1:1000 Cell Signaling | 1:10000 Cell Signaling | 1:15000 Cell Signaling |
| Loading Control | Lamin B1 | β−tubulin | β−tubulin | β−tubulin |

**Measures of Oxidative Stress**

Protein Carbonyls

50 mg of tissue powder was suspended in 500 ul of ice cold PBS + 0.5 mM EDTA buffer, gently mixed, and centrifuged at 10,000 rpm for 5 minutes at 4^0^C. The pellet was collected and suspended in 300 uL ice cold PBS + 0.5 mM EDTA buffer containing a protease and phosphatase inhibitor cocktail (Halt^TM^), followed by sonication on ice (5 cycles of 10 pulses, power 8, Fisher Scientific Sonic Dismembrator Model F60) over a total time of 15 minutes.

Samples were centrifuged at 13,000 rpm for 5 minutes at 4^0^C. 10% streptozocin solution was added to 30 uL of the supernatant and incubated at room temperature for 15 minutes in order to remove nucleic acids that would interfere with the protein carbonyl assay. Samples were then centrifuged again at 13,000 rpm for 5 minutes at 4^0^C. The supernatant was collected and used for a commercial protein carbonyl content assay. 5 uL of the sample was set aside for measurement of protein concentration using the Bicinchoninic Acid (BCA) assay.

Protein carbonyl content was measured using the Protein Carbonyl Content Assay Kit (MAK094, Sigma-Aldrich, St. Louis, MO) in accordance with the manufacturer’s instructions. This assay is based on the 2,4-dinitrophenylhydrazine (DNPH) reaction. DNPH was added to 100 uL of each sample which was then vortexed and incubated at room temperature for 10 minutes. TCA was then added followed by vortexing, incubation on ice for 5 minutes, centrifugation at 13,000g for 2 minutes (at -4C), and removal of supernatant. Acetone was added to the pellet which was sonicated and centrifuged. Guanidine solution was added and sonication repeated, after which absorbance was measured at 375 nm (A_375_). Carbonyl content was calculated based on A_375_ and indexed to protein content.

S-nitrosylated Proteins

Brain and Liver S-nitrosylation was analyzed using a S-nitrosylation Western blot kit (Thermo Scientific # 90105) according to the manufacturer’s instructions. Briefly, 50 mg of brain or liver grinded tissue powder was lysed in 500 ul of HENS buffer (100mM HEPES, pH 7.8; 1 mM EDTA; 0.1mM Neocuproine; 1% SDS) followed by sonication to reduce viscosity (2 x 10 pulses at power 8). The lysate was centrifuged at 10,000 rpm for 10 minutes in 4C, supernatant was collected, and protein concentrations were determined using BCA kit (Thermo Scientific).

2 µl of 1M MMTS was added to 100 µl of protein sample (200 µg protein in 100 µl of HENS buffer), and the sample was vortexed vigorously and incubated at room temperature for 30 minutes. To precipitate the protein, 600 µl of pre-chilled acetone was added to the samples and incubated at -20C for 1 hour. Samples were centrifuged at 10,000 rpm for 15 minutes in 4C and pellet was collected, air dried for 10 minutes and re-suspended in 100 ul of HENS buffer. To 50 ul of HENS buffer suspended samples 1 ul of labeling reagent (iodo TMT) and 2 ul of 1M sodium ascorbate were added, briefly vortexed and incubated at cold room overnight with constant agitation. The following day 600 µl of pre-chilled acetone was added to the samples which were incubated at -20C for 1 hour. Samples were centrifuged at 10,000 rpm for 15 minutes in 4C and pellet was collected, air dried for 10 minutes and re-suspended in 100 ul of HENS buffer.

To this iodo TMT-labelled sample (50 ul) 12.5 ul of 4X reducing Laemmli sample buffer was added and heated for 5 minutes at 99C. 20 ul of each sample was loaded onto 4 to 20% TGX gels. Proteins were transferred to PVDF membrane, blocked at room temperature for 1 hour in SuperBlock Blocking Buffer (Thermo-Fisher # 37515) and incubated at 4C overnight with anti-TMT antibody (1:2000, Thermo-Fisher # 90075). Membranes were washed three times in TBST for 10 minutes then incubated with anti-mouse IgG-HRP conjugated (1:15000) secondary antibody for 1h at room temperature. Immunoreactivity was detected using enhanced chemiluminescence substrate (Super Signal West Femto; Thermo Scientific). Images were captured using GBOX (Chemi XR5; Syngene), and gels were analyzed densitometrically using the computerized image analysis software (Gene Tools from Syngene). Loading controls (total protein) were membranes was washed and stained with Ponceau S solution.

**ARRIVE Guidelines**

Due to size constraints, not all elements of the ARRIVE guidelines are included in our manuscript. Additional details of our experimental methodology are included below to ensure compliance with ARRIVE:

All animals purchased from Envigo or Charles River.

Animals were housed in our institutional vivarium. Animals were paired and experienced 12 hour day-light cycles (6A-6P). They were given *ad libitum* access to food and water.

All animal experiments were performed from approximately 6:00 AM to 3:00 PM, in our laboratory.

No animals died during the 8 hours of anesthesia after sham-surgery or CLP.

**Additional file Referenced Statements**

(In the interest of keeping only the most essential references in the manuscript, we have provided additional citations that may be useful to the readership as part of a supplemental appendix; much of the background can be found in a review article recently published in *Anesthesia & Analgesia*^3^)

*Introduction*

Early demonstrations of preserved intracellular ATP levels in septic animal models initially suggested that mitochondrial function was unaltered by sepsis.^4-7^

Cells can adapt to decreased oxygen by decreasing ATP demand.^8,9^

Volatile anesthetic agents inhibit Complex I^10-14^

Volatile anesthetic agents lead to anaerobic metabolism and lactate production.^15^

Propofol does not appear to inhibit Complex I^16^ but may inhibit Complex II.^17,18^

*Methods*

Oxidized and reduced forms of cytochrome aa_3_ absorb near infrared radiation (NIR) differentially around 829 nm,^19^ it is possible to monitor the oxidation state of cytochrome aa_3_ (Cyt_OX_) in real time using near infrared spectroscopy (NIRS), in which the absorption of multiple wavelengths of light is used to calculate the concentration of various light-absorbing substances (chromophores) using linear algebraic techniques.^20^

Validated broadband NIRS algorithms^21-24^ (validated with cyanide injection)

Freeze-blow technique for instantaneous extraction of brain (and validation).^25,26^

*Discussion*

Halothane, isoflurane, and sevoflurane all inhibit Complex I of the ETC.^10-14^

Isoflurane has no observed effect on Complex II^13^, III^12,13^ or IV.^12,13^

Three magnetic resonance spectroscopy studies using rats have demonstrated increased cerebral lactate levels with isoflurane concentrations of 1.0 MAC or higher.^15,27,28^ Similar results have been obtained in monkeys exposed to isoflurane^29^ and humans exposed to sevoflurane.^30^

Reactive species including nitric oxide and peroxynitrate have been shown to directly inhibit Complex IV,^31 ,32,33^ as does carbon monoxide, a byproduct of the anti-oxidant protein HO-1. Volatile agents have been demonstrated to induce oxidative stress^34,35^ as well as induce HO-1 expression.^36,37^

Exposure of mitochondrial preparations to volatile anesthetic agents has revealed that halothane, isoflurane, and sevoflurane all inhibit Complex I of the ETC.^10-14^

NADH inhibits pyruvate dehydrogenase and is oxidized to NAD^+^ during the conversion of pyruvate to lactate via lactate dehydrogenase (LDH)^38^

Synthesis of data from multiple animal models of varying severity and time points suggests that mitochochondrial dysfunction (and accompanying energy failure) requires approximately 24 hours of sepsis exposure^3^ and similar results have been demonstrated in septic human surgical patients.^39-42^

Several authors have identified HIF-1 upregulation in the setting of exposure to endotoxin or sepsis^43-46^ but these studies did not report tissue oxygenation.

Several authors have identified HIF-1 upregulation in the setting of exposure to endotoxin or sepsis^43-46^ but these studies did not report tissue oxygenation.

Observations of non-hypoxic HIF-1 overexpression (during hyperbaric oxygen).^47-50^

**Additional file References**

1. Huang LE, Arany Z, Livingston DM, Bunn HF. Activation of hypoxia-inducible transcription factor depends primarily upon redox-sensitive stabilization of its alpha subunit. *J Biol Chem.* 1996;271(50):32253-32259.

2. Srinivasan S, Dunn JF. Stabilization of hypoxia-inducible factor-1alpha in buffer containing cobalt chloride for Western blot analysis. *Anal Biochem.* 2011;416(1):120-122.

3. Thiele RH. Subcellular Energetics and Metabolism: A Cross-Species Framework. *Anesthesia and analgesia.* 2017;124(6):1857-1871.

4. Hotchkiss RS, Song SK, Neil JJ, et al. Sepsis does not impair tricarboxylic acid cycle in the heart. *The American journal of physiology.* 1991;260(1 Pt 1):C50-57.

5. Hotchkiss RS, Long RC, Hall JR, et al. An in vivo examination of rat brain during sepsis with 31P-NMR spectroscopy. *The American journal of physiology.* 1989;257(6 Pt 1):C1055-1061.

6. Solomon MA, Correa R, Alexander HR, et al. Myocardial energy metabolism and morphology in a canine model of sepsis. *The American journal of physiology.* 1994;266(2 Pt 2):H757-768.

7. Levy RJ, Deutschman CS. Cytochrome c oxidase dysfunction in sepsis. *Crit Care Med.* 2007;35(9 Suppl):S468-475.

8. Schumacker PT, Chandel N, Agusti AG. Oxygen conformance of cellular respiration in hepatocytes. *The American journal of physiology.* 1993;265(4 Pt 1):L395-402.

9. Budinger GR, Duranteau J, Chandel NS, Schumacker PT. Hibernation during hypoxia in cardiomyocytes. Role of mitochondria as the O2 sensor. *J Biol Chem.* 1998;273(6):3320-3326.

10. Harris RA, Munroe J, Farmer B, Kim KC, Jenkins P. Action of halothane upon mitochondrial respiration. *Arch Biochem Biophys.* 1971;142(2):435-444.

11. Hanley PJ, Ray J, Brandt U, Daut J. Halothane, isoflurane and sevoflurane inhibit NADH:ubiquinone oxidoreductase (complex I) of cardiac mitochondria. *J Physiol.* 2002;544(Pt 3):687-693.

12. Kayser EB, Suthammarak W, Morgan PG, Sedensky MM. Isoflurane selectively inhibits distal mitochondrial complex I in Caenorhabditis elegans. *Anesthesia and analgesia.* 2011;112(6):1321-1329.

13. Hirata N, Shim YH, Pravdic D, et al. Isoflurane differentially modulates mitochondrial reactive oxygen species production via forward versus reverse electron transport flow: implications for preconditioning. *Anesthesiology.* 2011;115(3):531-540.

14. Pravdic D, Hirata N, Barber L, Sedlic F, Bosnjak ZJ, Bienengraeber M. Complex I and ATP synthase mediate membrane depolarization and matrix acidification by isoflurane in mitochondria. *Eur J Pharmacol.* 2012;690(1-3):149-157.

15. Makaryus R, Lee H, Yu M, et al. The metabolomic profile during isoflurane anesthesia differs from propofol anesthesia in the live rodent brain. *J Cereb Blood Flow Metab.* 2011;31(6):1432-1442.

16. Wu GJ, Tai YT, Chen TL, Lin LL, Ueng YF, Chen RM. Propofol specifically inhibits mitochondrial membrane potential but not complex I NADH dehydrogenase activity, thus reducing cellular ATP biosynthesis and migration of macrophages. *Ann N Y Acad Sci.* 2005;1042:168-176.

17. Kajimoto M, Atkinson DB, Ledee DR, et al. Propofol compared with isoflurane inhibits mitochondrial metabolism in immature swine cerebral cortex. *J Cereb Blood Flow Metab.* 2014;34(3):514-521.

18. Yu W, Gao D, Jin W, Liu S, Qi S. Propofol Prevents Oxidative Stress by Decreasing the Ischemic Accumulation of Succinate in Focal Cerebral Ischemia-Reperfusion Injury. *Neurochemical research.* 2018;43(2):420-429.

19. Wray S, Cope M, Delpy DT, Wyatt JS, Reynolds EO. Characterization of the near infrared absorption spectra of cytochrome aa3 and haemoglobin for the non-invasive monitoring of cerebral oxygenation. *Biochim Biophys Acta.* 1988;933(1):184-192.

20. Jobsis FF. Noninvasive, infrared monitoring of cerebral and myocardial oxygen sufficiency and circulatory parameters. *Science.* 1977;198(4323):1264-1267.

21. Cooper CE, Cope M, Springett R, et al. Use of mitochondrial inhibitors to demonstrate that cytochrome oxidase near-infrared spectroscopy can measure mitochondrial dysfunction noninvasively in the brain. *J Cereb Blood Flow Metab.* 1999;19(1):27-38.

22. Lee J, Armstrong J, Kreuter K, Tromberg BJ, Brenner M. Non-invasive in vivo diffuse optical spectroscopy monitoring of cyanide poisoning in a rabbit model. *Physiol Meas.* 2007;28(9):1057-1066.

23. Thiele RH, Ikeda K, Wang Y, Bartz RR, Zuo Z. Broadband near-infrared spectroscopy can detect cyanide-induced cytochrome aa3 inhibition in rats: a proof of concept study. *Can J Anaesth.* 2017;64(4):376-384.

24. Thiele RH, Ikeda K, Osuru HP, Zuo Z. Comparison of Broadband and Discrete Wavelength Near-Infrared Spectroscopy Algorithms for the Detection of Cytochrome aa3 Reduction. *Anesthesia and analgesia.* 2018.

25. Veech RL, Harris RL, Veloso D, Veech EH. Freeze-blowing: a new technique for the study of brain in vivo. *J Neurochem.* 1973;20(1):183-188.

26. Lust WD, Passonneau JV, Veech RL. Cyclic adenosine monphosphate, metabolites, and phosphorylase in neural tissue: a comparison a methods of fixation. *Science.* 1973;181(4096):280-282.

27. Pfeuffer J, Tkac I, Choi IY, et al. Localized in vivo 1H NMR detection of neurotransmitter labeling in rat brain during infusion of [1-13C] D-glucose. *Magn Reson Med.* 1999;41(6):1077-1083.

28. Tkac I, Rao R, Georgieff MK, Gruetter R. Developmental and regional changes in the neurochemical profile of the rat brain determined by in vivo 1H NMR spectroscopy. *Magn Reson Med.* 2003;50(1):24-32.

29. Valette J, Guillermier M, Besret L, Hantraye P, Bloch G, Lebon V. Isoflurane strongly affects the diffusion of intracellular metabolites, as shown by 1H nuclear magnetic resonance spectroscopy of the monkey brain. *J Cereb Blood Flow Metab.* 2007;27(3):588-596.

30. Jacob Z, Li H, Makaryus R, et al. Metabolomic profiling of children's brains undergoing general anesthesia with sevoflurane and propofol. *Anesthesiology.* 2012;117(5):1062-1071.

31. Bolanos JP, Peuchen S, Heales SJ, Land JM, Clark JB. Nitric oxide-mediated inhibition of the mitochondrial respiratory chain in cultured astrocytes. *J Neurochem.* 1994;63(3):910-916.

32. Cleeter MW, Cooper JM, Darley-Usmar VM, Moncada S, Schapira AH. Reversible inhibition of cytochrome c oxidase, the terminal enzyme of the mitochondrial respiratory chain, by nitric oxide. Implications for neurodegenerative diseases. *FEBS Lett.* 1994;345(1):50-54.

33. Sharpe MA, Cooper CE. Interaction of peroxynitrite with mitochondrial cytochrome oxidase. Catalytic production of nitric oxide and irreversible inhibition of enzyme activity. *J Biol Chem.* 1998;273(47):30961-30972.

34. Sedlic F, Pravdic D, Ljubkovic M, Marinovic J, Stadnicka A, Bosnjak ZJ. Differences in production of reactive oxygen species and mitochondrial uncoupling as events in the preconditioning signaling cascade between desflurane and sevoflurane. *Anesthesia and analgesia.* 2009;109(2):405-411.

35. Muravyeva M, Sedlic F, Dolan N, Bosnjak ZJ, Stadnicka A. Preconditioning by isoflurane elicits mitochondrial protective mechanisms independent of sarcolemmal KATP channel in mouse cardiomyocytes. *J Cardiovasc Pharmacol.* 2013;61(5):369-377.

36. Schmidt R, Tritschler E, Hoetzel A, et al. Heme oxygenase-1 induction by the clinically used anesthetic isoflurane protects rat livers from ischemia/reperfusion injury. *Annals of surgery.* 2007;245(6):931-942.

37. Li Q, Zhu Y, Jiang H, Xu H, Liu H. Up-regulation of heme oxygenase-1 by isoflurane preconditioning during tolerance against neuronal injury induced by oxygen glucose deprivation. *Acta Biochim Biophys Sin (Shanghai).* 2008;40(9):803-810.

38. Batenburg JJ, Olson MS. Regulation of pyruvate dehydrogenase by fatty acid in isolated rat liver mitochondria. *J Biol Chem.* 1976;251(5):1364-1370.

39. Brealey D, Brand M, Hargreaves I, et al. Association between mitochondrial dysfunction and severity and outcome of septic shock. *Lancet.* 2002;360(9328):219-223.

40. Liaw KY, Askanazi J, Michelson CB, Kantrowitz LR, Furst P, Kinney JM. Effect of injury and sepsis on high-energy phosphates in muscle and red cells. *J Trauma.* 1980;20(9):755-759.

41. Tresadern JC, Threlfall CJ, Wilford K, Irving MH. Muscle adenosine 5'-triphosphate and creatine phosphate concentrations in relation to nutritional status and sepsis in man. *Clin Sci (Lond).* 1988;75(3):233-242.

42. Fredriksson K, Hammarqvist F, Strigard K, et al. Derangements in mitochondrial metabolism in intercostal and leg muscle of critically ill patients with sepsis-induced multiple organ failure. *Am J Physiol Endocrinol Metab.* 2006;291(5):E1044-1050.

43. Scharte M, Han X, Uchiyama T, Tawadrous Z, Delude RL, Fink MP. LPS increases hepatic HIF-1alpha protein and expression of the HIF-1-dependent gene aldolase A in rats. *J Surg Res.* 2006;135(2):262-267.

44. Bateman RM, Tokunaga C, Kareco T, Dorscheid DR, Walley KR. Myocardial hypoxia-inducible HIF-1alpha, VEGF, and GLUT1 gene expression is associated with microvascular and ICAM-1 heterogeneity during endotoxemia. *Am J Physiol Heart Circ Physiol.* 2007;293(1):H448-456.

45. Seehase M, Gantert M, Ladenburger A, et al. Myocardial response in preterm fetal sheep exposed to systemic endotoxinaemia. *Pediatr Res.* 2011;70(3):242-246.

46. Oliveira-Pelegrin GR, Basso PJ, Rocha MJ. Cellular bioenergetics changes in magnocellular neurons may affect copeptin expression in the late phase of sepsis. *J Neuroimmunol.* 2014;267(1-2):28-34.

47. Peng Z, Ren P, Kang Z, et al. Up-regulated HIF-1alpha is involved in the hypoxic tolerance induced by hyperbaric oxygen preconditioning. *Brain Res.* 2008;1212:71-78.

48. Ren P, Kang Z, Gu G, et al. Hyperbaric oxygen preconditioning promotes angiogenesis in rat liver after partial hepatectomy. *Life Sci.* 2008;83(7-8):236-241.

49. Hu Q, Liang X, Chen D, et al. Delayed hyperbaric oxygen therapy promotes neurogenesis through reactive oxygen species/hypoxia-inducible factor-1alpha/beta-catenin pathway in middle cerebral artery occlusion rats. *Stroke; a journal of cerebral circulation.* 2014;45(6):1807-1814.

50. Sunkari VG, Lind F, Botusan IR, et al. Hyperbaric oxygen therapy activates hypoxia-inducible factor 1 (HIF-1), which contributes to improved wound healing in diabetic mice. *Wound Repair Regen.* 2015;23(1):98-103.
